# Electrolytes at Uncharged Liquid Interfaces: Adsorption, Potentials, Surface Tension, and the Role of the Surfactant Monolayer

**DOI:** 10.1021/acs.langmuir.4c01388

**Published:** 2024-08-12

**Authors:** Radomir Iliev Slavchov, Boyan Peychev, Ivan Minkov

**Affiliations:** † School of Engineering and Materials Science, Queen Mary University of London, London E1 4NS, United Kingdom; ‡ Rostislaw Kaischew Institute of Physical Chemistry, Bulgarian Academy of Sciences, 1113 Sofia, Bulgaria; § Department of Chemistry, Biochemistry, Physiology, and Pathophysiology, Faculty of Medicine, Sofia University, 1407 Sofia, Bulgaria

## Abstract

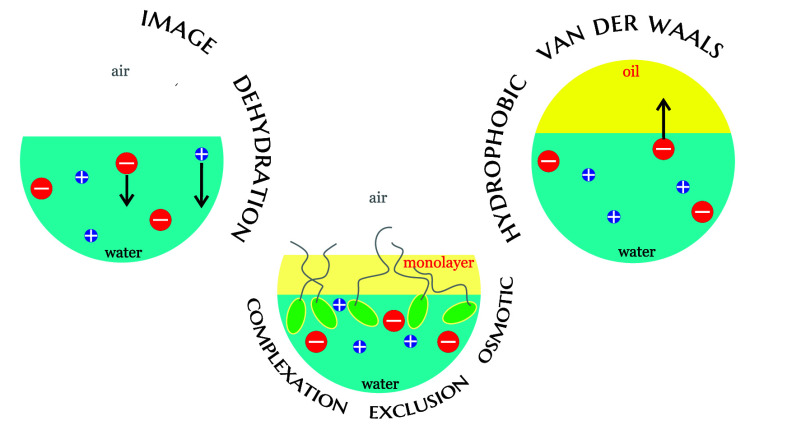

The article summarizes the results of our research on the behavior of ions at uncharged fluid interfaces, with a focus on moderately to highly concentrated aqueous electrolytes. The ion-specific properties of such interfaces have been analyzed. The ion-specificity series are different for water|air and water|oil; different for surface tension *σ*, surface Δ*χ* potential and electrolyte adsorption, and they change with concentration. A methodology has been developed that allows to disentangle the multiple factors controlling the ion order. The direct ion-surface interactions are not always the most significant factor behind the observed ion sequences: indirect effects stemming from conjugate bulk properties are often more important. For example, the order of the surface tension with the nature of the anion (*σ*_KOH_ > *σ*_KCl_ > *σ*_KNO_3__ for potassium salts) is often the result of bulk nonideality and follows the order of the bulk activity coefficients (*γ*_KOH_ > *γ*_KCl_ > *γ*_KNO_3__) rather than that of a specific ion-surface interaction potential. The surface Δ*χ* potential of aqueous solutions is, in many cases, insensitive to the ion distribution in the electric double layer but reflects the orientation of water at the surface, through the ion-specific dielectric permittivity *ε* of the solution. Even the sign of Δ*χ* is often the result of the decrement of *ε* in the presence of electrolyte. A whole new level of complexity appears when the ions interact with an uncharged surfactant monolayer. A method has been developed to measure the electrolyte adsorption isotherms on monolayers of varying area per surfactant molecule via a combination of experiments–compression isotherms and surface pressure of equilibrium spread monolayers. The obtained isotherms demonstrate that the ions exhibit a maximum in their adsorption on monolayers of intermediate density. The maximum is explained with the interplay between ion-surfactant complexation, volume exclusion and osmotic effects.

## Introduction

In 1887, Hofmeister discovered a regularity in the ability of various salts to precipitate proteins:^1^ the critical electrolyte concentration of protein precipitation decreases following an order of ions called nowadays the *lyotropic*, or *Hofmeister series*. For salts having the same cation and different anions, the precipitation efficiency increases in the following sequence:^2^
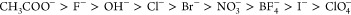
1

Similarly, for salts sharing the anion, the Hofmeister cation series reads^2^
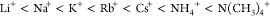
2

These sequences are approximately independent of the protein and the nature of the shared counterion, although the *direction* of the effect may depend on the sign of the protein’s net charge.^3,4^

Hofmeister’s results demonstrated that it is more than just the ion charge that governs the interaction of an electrolyte with the interface between aqueous solution and a relatively hydrophobic entity (like a protein). Since 1887, a long list of properties of such hydrophobic interfaces were shown to exhibit ion-specificity, often following to some extent the original Hofmeister series. Examples of commercial importance are the surface activity and micelle structure of ionic surfactants,^5,6^ the interaction of electrolytes with organics, stability of electrolyte-containing emulsions and foams in mining, wetting and adsorption on hydrophobic solids etc. The lyotropic series also has a biological significance (ions interacting with membranes, DNA, proteins;^7^ sea ecology) and may play a role in Earth’s climate.^8^

The understanding of the ion-specific phenomena exhibited at hydrophobic interfaces proved to be difficult even for the simplest systems. It is now known that many distinct ion-specific effects exist. The Hofmeister series (eqs 1 and 2) give a rough guide but are rarely followed exactly. Consider the following experimental findings:

- surface tension at water|air (W|A),^9−11^
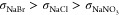
3

- interfacial tension at water|hydrocarbon (W|H),^12−14^
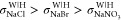
4

- surface potential at W|A, 5 mol/kg,^15^
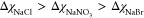
5

- surface potential at W|A, 2 mol/kg,^15^
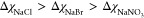
6

These examples show that the series vary with the studied system: the surface tension *σ* of electrolyte solutions follows a different order from that of the interfacial tension *σ*^W|H^ of aqueous electrolyte|alkane, eqs 3 and 4. Even for the same system, the series vary with the property considered: at W|A, the order followed by the surface Δ*χ* potential is not the same as that of *σ*, eqs 3 and 5. Even for the same system and property, the order changes with concentration: the Δ*χ* series is different at 2 mol/kg and 5 mol/kg, eqs 5 and 6. This demonstrates that the ion-specificity is controlled by numerous competing factors: to begin with, a long list of specific ion-surface interactions, but also, the measured surface property is often ion-specific because of an underlying bulk ion-specific phenomenon. Therefore, the first necessary step toward the understanding of these phenomena is classification.^16^

**Figure 1:**
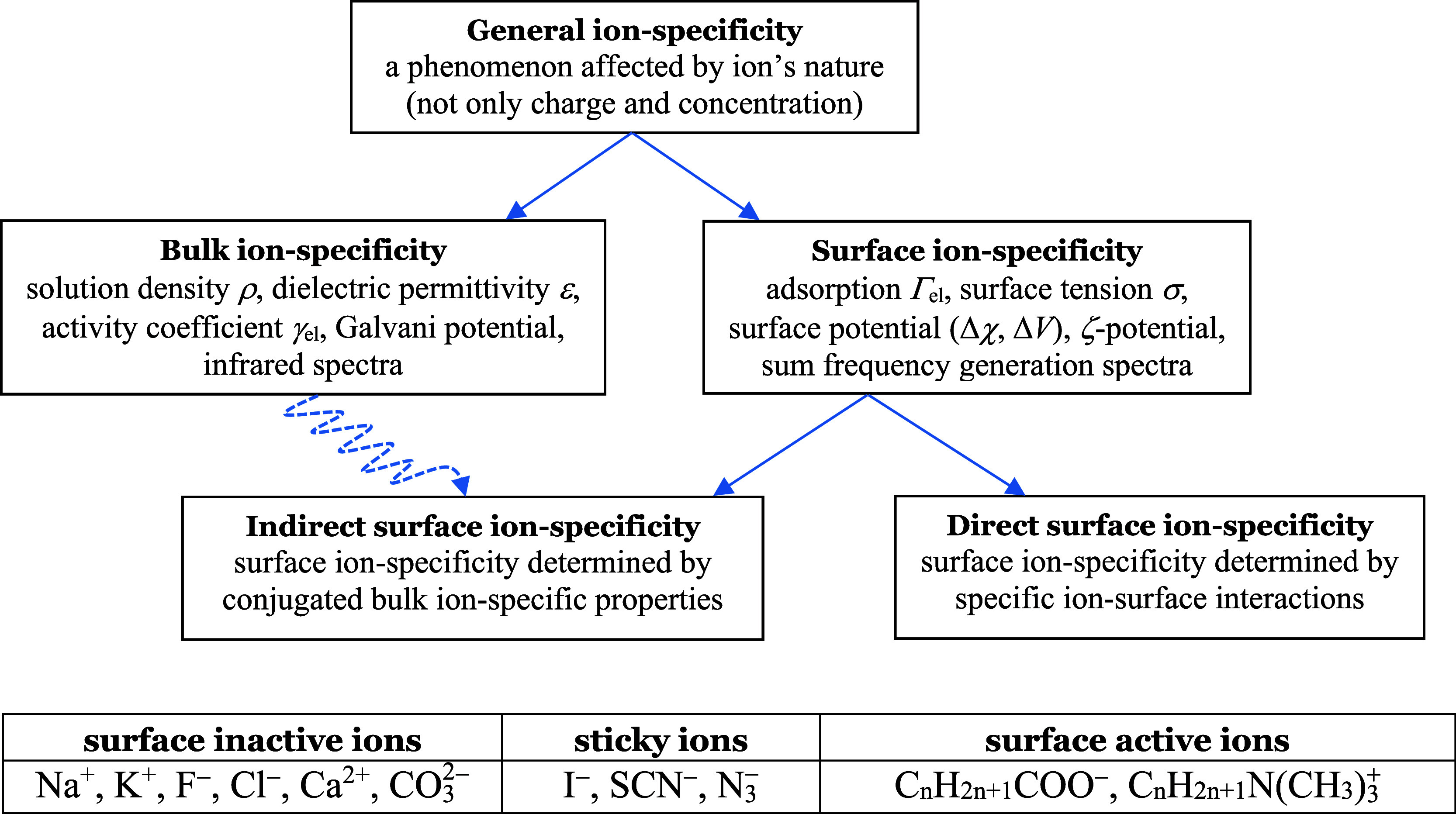
Classification of the ion-specific phenomena and of the ions by their affinity to hydrophobic surfaces (based on ref (17)).

It appears useful to split the surface ion-specific effects into two classes: direct and indirect^17^ (see Figure 1). By *direct surface ion-specificity* we indicate a phenomenon controlled by a specific interaction potential between the ion and the studied interface; for instance, the surface tension of aqueous NaI is lower than that of NaCl because I^–^ is attracted to the surface. Often, however, a surface property is ion-specific because of conjugation with a respective bulk property; for example, the surface tension *σ* of NaBr is higher than that of NaNO_3_ because the bulk activity coefficient *γ*_el_ of NaBr is higher, and *σ* and *γ*_el_ are related through the Gibbs isotherm. We call this phenomenon *indirect surface ion-specificity*.

Another useful classification is of the ions themselves. The first category contains small monovalent and most multivalent inorganic ions, which tend to exhibit relatively weak direct surface specificity. Salts of such ions still manifest ion-specific properties at hydrophobic surfaces, but these are dominated by indirect effects: the surface tension of their solutions follows the ion order of the bulk activity coefficient, while their surface potential follows the one of the bulk dielectric constant.^9,18,12^ In contrast, bulky monovalent ions such as I^–^ show a clear tendency to adsorb to hydrophobic surfaces and are therefore classified as sticky.^19,17^ The ionic surfactants form a third class.

In this feature article, we summarize the results of our group for a particular class of systems featuring ions at interfaces: namely, interfaces that carry no intrinsic charge (W|A, W|H, water|uncharged surfactant monolayer|air) at intermediate to high electrolyte concentration (0.1–10 M), focusing on the thermodynamic properties of the boundary (interfacial tension, electrolyte adsorption) and its electrical properties (Δ*χ* and Δ*V* potentials). Vast amount of thermodynamic data has been accumulated for this class of systems,^10,11^ which allows for some generalizations. Nevertheless, the level of disagreement about even the basic questions around the ion-specific effects in these systems is unprecedented in the field of physical chemistry. Authors in the field disagree on questions such as, which forces are causing the affinity of some ions to the surface? What is the direction of these forces? What is the reason for the sign of Δ*χ*? Which ion adsorbs more at W|A, Cl^–^ or Br^–^?

We will also attempt, where possible, to bridge the knowledge about ions at neat interfaces and ions adsorbing on surfactant-covered surfaces. Finally, we will try to define better the boundary between surface inactive and sticky ions, and the direct and indirect ion-specificity.

## Ions at Surfactant-Free W|A and W|H

The ion-specific effects on *σ*, Γ_el_ and Δ*χ* of electrolyte solutions are often comparable in magnitude to the uncertainty of the experimental data, especially at *C*_m_ < 1 mol/kg. On the other hand, the magnitude of these effects is the result of a fine balance between several competing factors. Therefore, a theory that oversimplifies the description of even one factor (dispersion interaction, bulk nonideality, image forces...) does that at the cost of a much-reduced range of validity. However, developing a general theory of the ion specificity would involve fiddling with many uncertain parameters.

In view of this, we adopted a somewhat unusual approach to investigate the Hofmeister series: rather than looking for a detailed theory involving all relevant interactions (dispersion, hydrophobic, ion-surface dipole) and trying to work out a parametrization for them, we instead have chosen a “minimal model” that takes into account all better established and ubiquitous effects (image and dehydration forces, double layer, electrolyte activity), and we analyzed the deviations between the experimental data and this model to characterize the “missing” interactions. The model we chose was developed by Schmutzer;^20^ it combines the dehydration and image interactions (known to contribute to the ion adsorption since Langmuir and Onsager) with direct account for the electrolyte activity. We complemented it with an explicit model for the dehydration interaction and a standard electric double layer (EDL) description; the resulting *modified Schmutzer’s model* (SchM) was then compared to all the tensiometric data we could find.

Surprisingly, this minimal model turned out to be predictive for a whole class of electrolytes–namely, all combinations of surface inactive ions, including monovalent^9^ and multivalent ions^18^ and water|oil interfaces;^12^ we even applied it successfully to explain Δ*χ* potential data.^18^ Equally interesting is the use of SchM where it deviates from the measurements: we were able to utilize the model as a tool to subtract the “trivial” indirect effects and the contributions from quantitatively understood forces from the experimental data for sticky ions. The deviations between SchM and tensiometric and potentiometric data are strongly correlated to basic ion properties and allow to draw important conclusions about the nature, direction, and magnitude of the ion-surface forces.

### Surface(s) of Water

**Figure 2:**
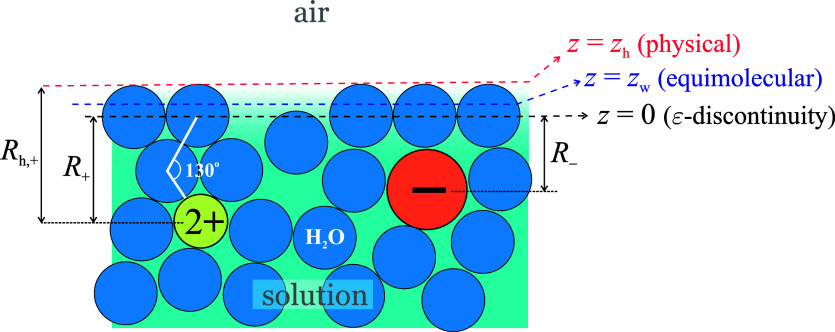
Structure of the surface layer of an aqueous electrolyte solution. The image force acting on the ions depends on the distance to the dielectric surface (*z* = 0); the dehydration potential keeps the hydrated ions (of radius *R*_h,*i*_) away from the physical surface (*z*_h_). Both are different from the equimolecular surface (*z*_w_). Adapted with permission from ref (18). Copyright 2013 Elsevier.

The boundary between an electrolyte solution and air is a three-dimensional object (Figure 2). The main structural features of this layer can be characterized with the location of three surfaces:^12^

#### Dielectric Surface or Surface of *ε*-Discontinuity (*z* = 0)

This is the surface where the excess of the dielectric permittivity *ε* is zero. In fact, in the anisotropic surface layer, the dielectric permittivity *ε* is a tensor and its excess has both normal and tangential components. The lateral components produce a tangential surface polarizability which affects the state of charges at the surface.^21,22^ Moreover, the surface possesses an excess of normal polarization–i.e., a surface dipole moment,^23,24^ even in the absence of electric field. This complexity allows for several possible reasonable choices of the location of the dielectric surface. Within our minimal model, we assume that the trace of the *ε* tensor is approximately proportional to the density of the surface layer, and we identify the *ε*-discontinuity with the discontinuity of the density of the solution—i.e., the location of the *ε*-surface is within the topmost sparse layer of water molecules (Figure 2).

#### Physical Surface (*z* = *z*_h_)

If water is brought in contact with an ideal noninteracting wall, this is the surface of closest possible approach of the wall to the surface layer, which is located approximately at the top of the last dense layer of water molecules (Figure 2). The distance *z*_h_ between the physical and the *ε*-discontinuity surfaces reflects the reduced density of water in the surface layer and is known as the *thickness of the hydrophobic gap*. It has been measured directly via X-ray reflectivity^25^ for water in contact with a nearly ideal hydrophobic solid, and its value was found to be *z*_h_ = 1.38 Å, corresponding roughly to a surface made up of a single layer of water molecules of density reduced by 50% compared to the bulk.

#### Gibbs Equimolecular Surface of Water (*z* = *z*_w_)

This is the surface where the excess Γ_w_ of the water concentration is zero. The location of the equimolecular surface *z*_w_ is a natural result of every adsorption model and can be related directly to the adsorption of electrolyte and surfactant. For pure water, it coincides with the dielectric surface (i.e., *z*_w_ = 0). However, the presence of a concentrated electrolyte would usually diminish the bulk concentration of water, while the surface layer (where the ions are depleted) remains approximately unchanged compared to pure water.^26^ As a result, an excess of water appears at *z* = 0, shifting the equimolecular surface toward the air by ∼0.05 Å per mol/kg of electrolyte.

The structure of the surface layer is an essential element of the ion-surface interaction because each surface force “sees” a different surface. The image force is caused by the discontinuity of *ε*; therefore, the distance appearing in the image force expression of Onsager and Samaras^27^ is distance to the dielectric surface *z* = 0. The dehydration force is usually modeled as a hard-wall interaction, and the distance appearing in the hard-wall expression is a distance to the physical surface of water (*z* = *z*_h_). Moreover, the Gibbs isotherm posits that the derivative of the surface tension with respect to the electrolyte activity gives the electrolyte adsorption at the equimolecular surface of water (*z* = *z*_w_).

### Ion-Surface Interactions and Structure of the EDL

#### Screened Image Force

The image potential *u*_im_ is produced by the interaction between the ion charge and the solvent static polarizability. In concentrated electrolytes, *u*_im_ is screened, as realized first by Wagner.^28^ Wagner’s semiempirical expression, in a simplified form, was derived theoretically by Onsager and Samaras;^27^ their result for the image potential acting on the *i*^th^ ion vs the distance *z* to the *ε*-discontinuity surface at W|A or W|H reads:
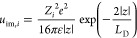
7Here, *L*_D_ is the Debye length defined with the equation:
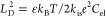
8*e* is elementary charge; ε is absolute dielectric permittivity of the aqueous solution; the coefficient *k*_is_ = (ν_+_Z_+_^2^ + ν_–_Z_–_^2^)/2 is the ratio between ionic strength and electrolyte concentration *C*_el_; ν_*i*_ is the stoichiometric number of the *i*^th^ ion and Z_*i*_ is its valence (full list of symbols provided in the supplement).

Onsager and Samaras used eq 7 to derive the limiting law for the adsorption of electrolyte Γ_el_ and the surface tension *σ*. They demonstrated that the long-range of the image repulsion results in the absence of a Henry region in the electrolyte adsorption isotherm,^27^ i.e., rather than a linear dependence on *C*_el_ of Γ_el_ and Δ_el_*σ* (as for any uncharged solute at infinite dilution), a stronger limiting dependence on *C*_el_ is followed at *C*_el_ → 0, namely Δ_el_*σ* ∝ *C*_el_·ln*C*_el_.

The image force is charge-specific but not ion-specific. Some indirect dependence on the nature of the electrolyte enters *u*_im,*i*_ through ε, since concentrated electrolyte solutions have ion-specific dielectric permittivity. This effect, however, was found to be small.^9^ Onsager and Samaras^27^ considered an ion-specific volume exclusion effect (to make their model a full surface analogue to the extended Debye–Hückel theory), but this correction is negligible.^9^ Moreover, the image force is practically independent of whether the hydrophobic phase is oil or air. A correction factor (*ε* – *ε*^H^)/(*ε* + *ε*^H^) is sometimes added to eq 7 to account for the permittivity *ε*^H^ of the hydrophobic phase,^29^ but, since *ε* ≫ *ε*^H^ for the systems we consider, this factor is almost equal to one.

#### Dehydration Potential

The continuum image force model for the charge-dipole interaction breaks for the last few ångstroms of distance between the ion and the surface due to structural effects, i.e., to stepwise dehydration of the ion. We call the closest-neighbors correction of the continuum image force the *dehydration interaction* (here we use the term “dehydration”, rather than “hydration” potential as previously,^9^ to distinguish the phenomenon from the distinct hydration surface force^30^). The model for this short-ranged interaction postulated by Schmutzer and many others^20,31,32^ is that the water molecules in the first hydration shell of the ion are so strongly bound that the ion and its shell behave as a single particle of radius *R*_h,*i*_, interacting with the surface as if it is a solid wall. The surface in question is the physical surface of water;^18^ therefore, the solid-wall dehydration potential is
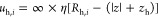
9where η is the Heaviside stepwise function. Consequently, the depletion layer of a hydrated ion of radius *R*_h*,i*_ spreads to a distance *R*_*i*_ away from the dielectric surface *z* = 0, smaller than *R*_h,*i*_ by the thickness of the hydrophobic gap:
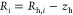
10

The parameter *R*_*i*_ enters in some form or another most theories of the surface specific effect and is typically the most sensitive parameter for the prediction of the adsorption of electrolyte. *R*_*i*_ is usually “adjusted” to match the observations; instead, we used data for the structure of the hydration shell to calculate it directly.^18^ The hydrated ionic radius depends on how strongly the water molecule is bound to the ion. For all monovalent ions, it appears that only the first hydration shell is bound strongly enough to produce a hard-wall potential; therefore, the hydrated ion radius is found as^33^
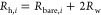
11where *R*_bare,*i*_ is the crystallographic radius of the ion (we used the values from ref (34)) and *R*_w_ = 1.38 Å is the Van der Waals (VdW) radius of a water molecule. Thus, the range of the dehydration force of all monovalent ions is calculated as
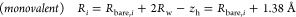
12

The polyvalent ions are more strongly hydrated: the second peak of the ion–water correlation function of a divalent ion is of height similar to that of the first peak for a monovalent ion.^35,36^ Besides, the precise location of the second hydration shell depends on its structure. For many ions,^36^ the angle between the ion, a water molecule from the first hydration shell, and water from the second hydration shell is approximately 130° (Figure 2); the law of cosines then allows to find *R*_h,*i*_ of multivalent ions.^12^ The final result is well-approximated by^12^
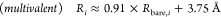
13

The dehydration force produces a direct ion-specific effect since *R*_bare,*i*_ is characteristic of the ion. This ion-specificity is relatively weak, e.g., for Li^+^ and Cs^+^, *R*_bare,*i*_ = 0.69 and 1.7 Å, respectively, but this difference translates in only 15–20% increase in the desorption of CsCl compared to LiCl in the molality range *C*_m_ = 1–5 mol/kg, and less below 1 mol/kg. Also, *u*_h_ does not change if the air is substituted with a nonpolar oil.

#### Electric Double Layer

Schmutzer’s original theory assumes that the depletion layers of both ions are of the same size, *R*_+_ = *R*_–_ (i.e., cation and anion of the same hydrated radius), which is reasonable for the electrolytes he considered. However, for two ions of dissimilar sizes, the difference in the dehydration potential produces surface charging and, respectively, formation of an ion diffuse layer. We modified Schmutzer’s model to allow for that. For the structure of the EDL, we adopted the common three-layer model.^37,38^ Using Na_2_CO_3_ solution as an example, where *R*_–_ > *R*_+_, the three layers are

(i) a top *ion-free layer* (|*z*| < *R*_+_), where both ions are depleted;
(ii) *ion adsorption layer* (*R*_–_ > |*z*| > *R*_+_), which is positively charged by the smaller Na^+^ ion, while the divalent CO_3_^2–^ is depleted due to stronger hydration;
(iii) *diffuse ion layer* (|*z*| > *R*_–_) which accumulates an excess of the counterion CO_3_^2–^ to neutralize the charge of the adsorption layer.

**Figure 3:**
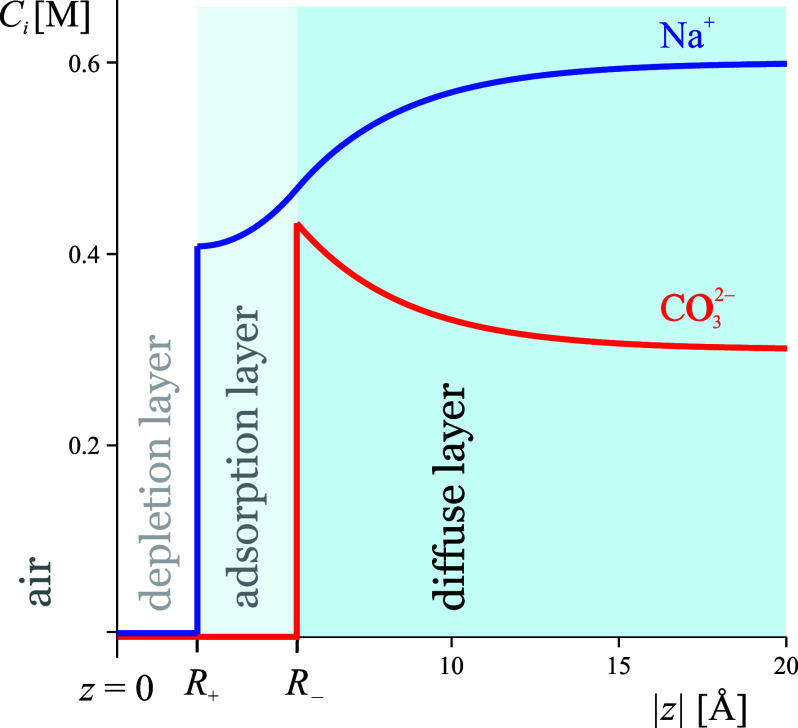
Ion concentration profiles in the EDL for 0.3 M Na_2_CO_3_. The surface charge carried by the adsorption layer is due to different dehydration interaction of the two ions with the surface. The adsorbed charge is compensated by the oppositely charged diffuse layer. Adapted with permission from ref (18). Copyright 2013 Elsevier.

Unlike the classical EDL theory, in concentrated electrolytes, the adsorption and the diffuse layer are of similar thicknesses (|*R*_–_ – *R*_+_| ∼ *L*_D_), due to strong screening. For the same reason, the EDL potential *ϕ*^DL^ is smaller than 15 mV, which allows linearization of the Boltzmann distribution, leading to analytic results for the potential and concentration profiles.^18^Figure 3 illustrates the ion distribution following from the linearized Poisson–Boltzmann equation. The formation of EDL contributes to both the surface potential and the adsorption of the salt.

The charging in this modification of Schmutzer’s model is due to the dehydration potential alone. For asymmetric electrolytes, additional charging due to the image force appears but is relatively small.^18^

#### Specific Interactions

The additional interactions that hypothetically should affect the adsorption of electrolytes (and are neglected in our minimal model) are the following ones:

##### Hydrophobic or Cavitational Attraction

Levin et al.^39,40,31^ have put forward a theory that explains the stickiness of larger ions such as I^–^ and SCN^–^ with the hydrophobic effect–the same force which expels the alkyl chain of surfactants out of the aqueous solution. Clearly, ions like N(CH_3_)_4_^+^ and CH_3_COO^–^ are driven to the surface by the hydrophobic force, so there can be no doubt that it contributes to the behavior of sticky ions.

The source of the hydrophobic effect is the entropic cost for the formation of hydration shell around large particles,^41^ related to reorganization of the hydrogen bond network with loss of proton disorder. A feature of the hydrophobic force is that its magnitude is proportional to the size (volume or area) of the particle.^40−42^ Moreover, the free energy gained from restoring the hydrogen bond network in the bulk when the ion is expelled at the surface of the solution should not depend on the nature of the hydrophobic phase.^40^ The shape of the hydrophobic potential is difficult to predict even for neutral particles, and the strong electric field of the ion should alter the range and magnitude of the hydrophobic effect; while we are not aware of investigations on this effect, that the dielectric saturation matters is clear from the fact that large divalent anions show no observable attraction to the surface.^18^

##### Van der Waals Forces

The Van der Waals interactions were highlighted as a major factor behind the Hofmeister effect at hydrophobic surfaces by Ninham and Yaminsky.^43^ This has been further explored by Ninham et al. and other authors.^44−47,40^ The static polarizability of the ions emerged as an essential surface ion-specific ingredient in the molecular dynamics simulation (MD) work by Jungwirth and Tobias.^48,49^

Let us remind briefly that the ion–solvent VdW potential has the following main components:^50^ (a) Debye force, i.e., ion polarizability-solvent static polarizability (dominated by water’s permanent dipole); (b) London dispersion force, i.e., high-frequency ion polarizability-solvent polarizability; (c) for ions carrying a dipole moment, the Keesom force is acting, i.e., an ion dipole-water dipole (dipole image force), and an additional Debye component for the ion dipole-solvent polarizability (which will be discussed separately below). The image potential may also be viewed as a kind of VdW interaction (longer-ranged ion charge-solvent static polarizability). The nonspecific ion charge-water permanent quadrupole interaction must be of similar range to (a-c)^51^ but has not been studied yet.

The VdW ion-surface force is understood even less than the hydrophobic interaction. This force is proportional to the excess polarizability, *α*_*i*_ – *α*_w_, over a range of frequencies, of the ion with respect to the water molecules it displaces when adsorbed,^43,45^ which is difficult to predict. There is no consensus in the literature even about whether the ion-surface VdW force is attractive of repulsive. To illustrate this important point, let us consider Br^–^ (*R*_bare,*i*_ = 1.96 Å, static polarizability *α*_*i*_/4πε_0_ = 4.85 Å^3^^34^). The transfer of Br^–^ from the aqueous solution to the surface is accompanied by a transfer of an ensemble of water molecules back into the aqueous solution, of static polarizability^52^*α*_w_/4πε_0_ = (*ε*_w_ – ε_0_)/(*ε*_w_ + 2ε_0_) × *R*_bare,*i*_^3^ = 7.25 Å^3^. The excess static polarizability of the ion is negative, (*α*_*i*_ – *α*_w_) /4πε_0_ = −2.4 Å^3^, meaning that the Debye contribution to VdW pushes the ion to the surface (the reason why Levin’s static formula^39^ is attractive). On the other hand, the dispersive component is controlled by the high-frequency polarizabilities; here, water’s dipole moment is irrelevant, i.e., the refractive index of water (*n*_w_ = 1.33) controls *α*_w_: *α*_w_/4πε_0_ = (*n*_w_^2^ – 1)/(*n*_w_^2^ + 2) × *R*_bare,*i*_^3^ = 1.55 Å^3^. The ion’s polarizability *α*_*i*_ is approximately unchanged at high frequency, so (*α*_*i*_ – *α*_w_) /4πε_0_ = +3.3 Å^3^; therefore, the excess London dispersion is a repulsive surface force. By using different assumptions for the dielectric saturation in the first hydration shell (completely orientated water molecules do not contribute to the Debye force), the density of the surface layer, the electrostrictive compression of the hydration shell,^45^ the screening of the Debye force, the effect of the electrolyte on *ε* etc., one can reverse both the sign of the VdW force and the direction of its dependence on the size of the ion. Ninham et al.^44^ settled for a choice producing repulsion pushing the ion to the bulk; Ivanov et al.^45^ went for attraction; Levin et al. took into account the Debye contribution but neglected the dispersion component for the ion–water interaction (but then introduced dispersion for ion-oil);^40^ Karraker and Radke had dispersion only;^47^ Jungwirth et al.^49^ demonstrated that a polarizable field pushes the ions to W|A (confirming the static effect considered by Levin et al.) but did not investigate whether their potentials have the right balance between London and Debye interactions.

Whatever the case, our “minimal model” approach allows to test two distinctive features of the VdW interactions. First, if two ions are of the same size but different polarizability (e.g., Ag^+^ vs K^+^), the more polarizable ion should exhibit stronger repulsion from W|A (while the contribution of the displaced water to VdW is unchanged). Second, in contrast to the hydrophobic and Debye interactions, the dispersion force is very sensitive to the nature of the second phase; e.g., since water and alkanes have similar Hamaker constants,^50^ the dispersion interaction should be “switched off” when air is substituted with alkane.

##### Other Effects and Interactions

There are numerous other factors that can contribute to the interaction of certain ions with the surface, which are specific to only a few ions and are therefore discussed in the following sections, upon comparison with experimental data.

## Electrolytes at W|A and W|H: Interfacial Tension

### Adsorption of the Electrolyte According to SchM

The substitution of the combination of dehydration, image, and double layer electrostatic potentials into the Boltzmann distribution and subsequent integration leads to the adsorption of the electrolyte. The result is well-approximated by the following formula^18^
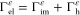
14where the image force contribution is given by
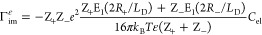
15and the dehydration force contribution, in the case where the anion is larger than the cation, if *R*_–_ > *R*_+_, is
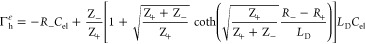
16

In the opposite case, where *R*_–_ < *R*_+_, the indices “+” and “–” in eq 16 must change place. E_1_ is the exponential integral. The “*ε*” superscript indicates that the adsorption is excess concentration with respect to the surface of dielectric discontinuity (*z* = 0 in Figure 2). This surface does not coincide with the Gibbs equimolecular one: since ions are depleted at the surface, a surface excess of water appears on *z* = 0. Water’s excess can be computed from the ion profiles,^9,18^ and is approximately given by
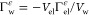
17*V*_el_ and *V*_w_ are the partial molecular volumes of the electrolyte and water, respectively.

The adsorption of water at *z* = 0 is directly related to the location *z*_w_ of the equimolecular surface, as follows from its Gibbs definition
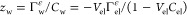
18where *C*_w_ = (1–*V*_el_*C*_el_)/*V*_w_ is the bulk concentration of water in the electrolyte solution. For positive *V*_el_ and negative electrolyte adsorption, the excess of water is positive at *z* = 0 and the shift *z*_w_ is toward the air phase; the opposite is true for the few electrolytes of negative *V*_el_.

Knowing the shift *z*_w_ allows one to calculate the Gibbs adsorption of the electrolyte^9,18^ as Γ_el_ = Γ_el_^*ε*^ – *z*_w_*C*_el_ at *z* = *z*_w_; this leads to
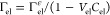
19

For neat electrolyte surfaces, the difference between Γ_el_^*ε*^ and the Gibbs adsorption Γ_el_ is significant only for few electrolytes of large *V*_el_ at high concentration. However, we will demonstrate below that the distinction becomes important when surfactant is present at the surface.

### Surface and Interfacial Tension

The surface tension follows from the integral of the Gibbs isotherm:
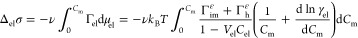
20Here, ν = ν_+_ + ν_*–*_ is the isotonic coefficient of the electrolyte and Δ_el_*σ* ≡ *σ* – *σ*_w_ is the surface tension increment upon adding electrolyte to pure water of surface tension *σ*_w_; eq 19 was used for Γ_el_ and eq 14 for Γ_el_^*ε*^; the chemical potential *μ*_el_ of the electrolyte was expressed through the mean molality-based activity coefficient *γ*_el_.^53^ This integral is explicit–the two contributions to the electrolyte adsorption are given by eqs 15 and 16. The evaluation of the integral (eq 20) requires a relationship between the activity *γ*_el_ and the molality *C*_m_ and also between the molarity *C*_el_ and *C*_m_. For these, we use experimental data.^9,12,18^ Two bulk ion-specific quantities appear in eq 20: the activity coefficient and the electrolyte density (the latter controls the dependence of the molarity *C*_el_ on the molality *C*_m_ and also *z*_w_). This introduces an indirect ion-specific effect in Δ_el_*σ*, i.e., the surface tension can be ion-specific even when the ion-surface forces and Γ_el_ are not.

SchM is insensitive to the nature of the hydrophobic phase^12^ – it predicts the exact same adsorption and interfacial tension at W|A and W|H, except for the case where the oil contains a polar headgroup that can penetrate the depletion layer.

### Comparison with Tensiometric Data for Surface Inactive Ions

**Figure 4:**
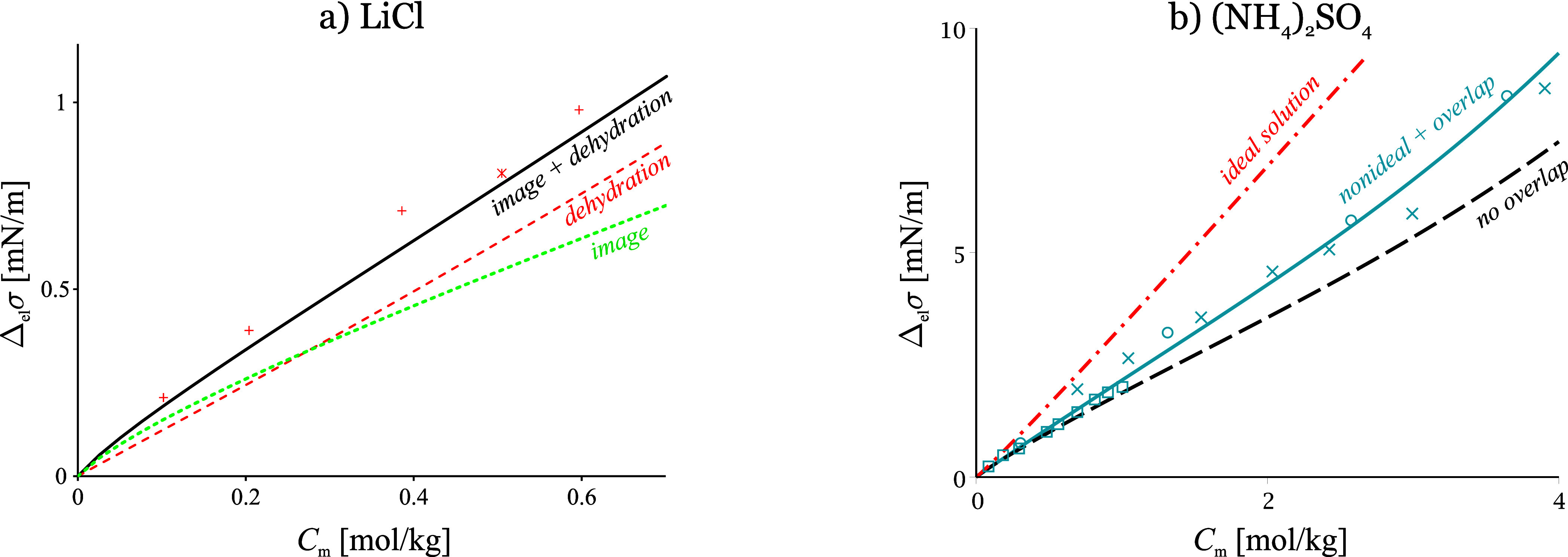
a) Contributions of the main interactions to the surface tension increment of aqueous LiCl. The “image + dehydration” line corresponds to SchM, eq 20; “image” corresponds to neglected dehydration force contribution (the Onsager-Samaras model); “dehydration” ignores the image force contribution. Adapted with permission from ref (9). Copyright 2012 Elsevier. b) Contribution of the overlap between ion diffuse and adsorption layers and of the nonideality to the surface tension increment of (NH_4_)_2_SO_4_. Adapted with permission from ref (18). Copyright 2013 Elsevier. Data points from refs (10), (13), (15), and (58).

All *surface inactive ions at W|A* follow the minimal model surprisingly accurately, and in a wide concentration range. A few examples are illustrated in Figure 4–Figure 7. SchM predicts Δ_el_*σ* for nearly any combination of a surface inactive cation (Li^+^, Na^+^, K^+^, Rb^+^, Cs^+^, NH_4_^+^, divalent Mg^2+^, Ca^2+^, Sr^2+^, Ba^2+^, trivalent Cr^3+^ and La^3+^) and a surface inactive anion (OH^–^, F^–^, Cl^–^, BrO_3_^–^, IO_3_^–^ ions that are often classified as sticky, like Br^–^ and NO_3_^–^; divalent SO_4_^2–^, CO_3_^2–^, CrO_4_^2–^), up to several mol/kg. While this agreement does not necessarily mean that small ions do not interact specifically with W|A, it certainly means that the tensiometric data are not sensitive to such interactions, either because they are small, or they cancel each other out. It is also notable that the list of ions that follow SchM is exactly the same as the α-ions of Craig et al.,^54^ a classification based on the effect of electrolytes on the coalescence of bubbles, possibly due to the d*σ*/d*C*_el_-driven Marangoni effect.^55−57^

**Figure 5:**
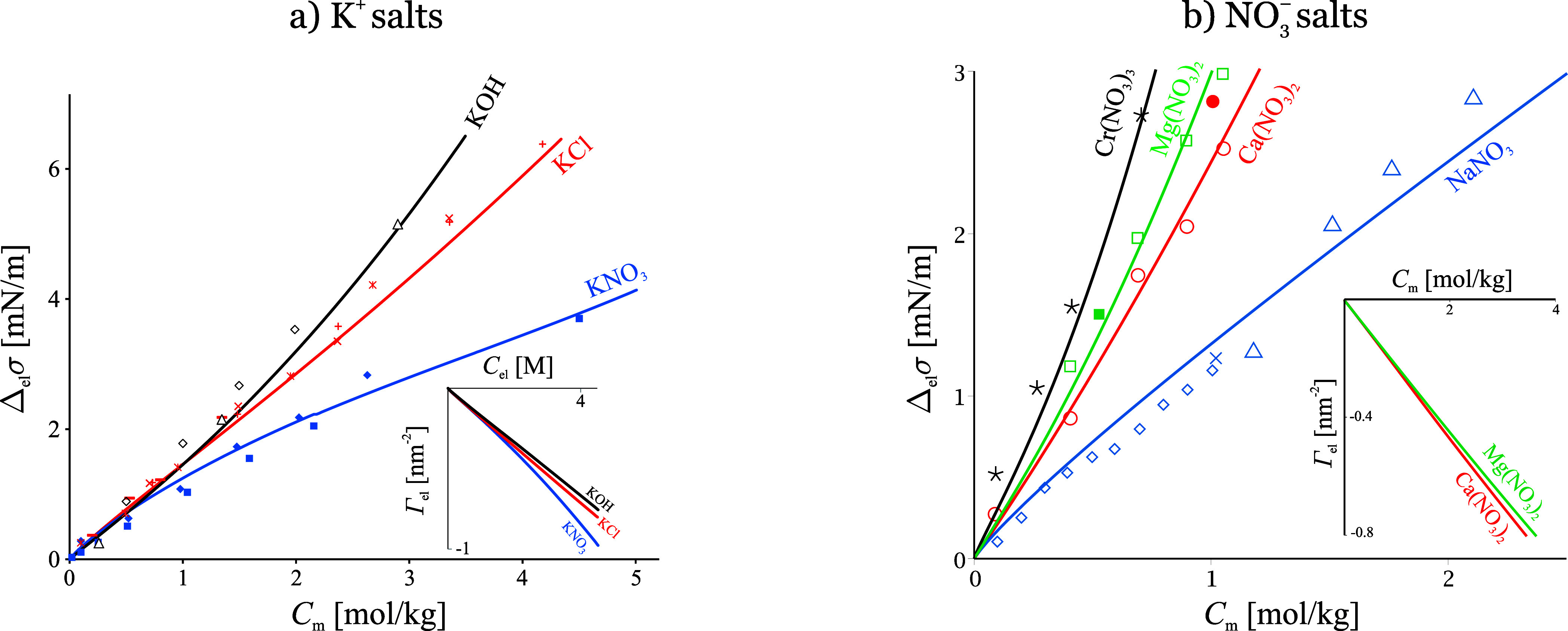
a) Indirect anion specificity: tensiometric data for potassium salts^13,10^ vs eq 20 of SchM. The surface tension follows the order of the specific activity coefficient values of KOH, KCl and KNO_3_. The respective desorptions (shown in the inset) follow the opposite order from what the surface tension of an ideal solution would suggest. Adapted with permission from ref (9). Copyright 2012 Elsevier. b) Charge specificity and indirect cation specificity for nitrates: data^15,10,70,71^ vs eq 20. The difference between Na^+^, Ca^2+^ and Cr^3+^ is due to the isotonic coefficient and the stronger hydration of Ca^2+^ and Cr^3+^. The surface tension of Mg(NO_3_)_2_ is higher than that of Ca(NO_3_)_2_ because γ_Mg(NO_3_)_2__ > γ_Ca(NO_3_)_2__; Mg^2+^ actually desorbs less than Ca^2+^ (inset). Adapted with permission from ref (18) and corrected. Copyright 2013 Elsevier.

**Figure 6:**
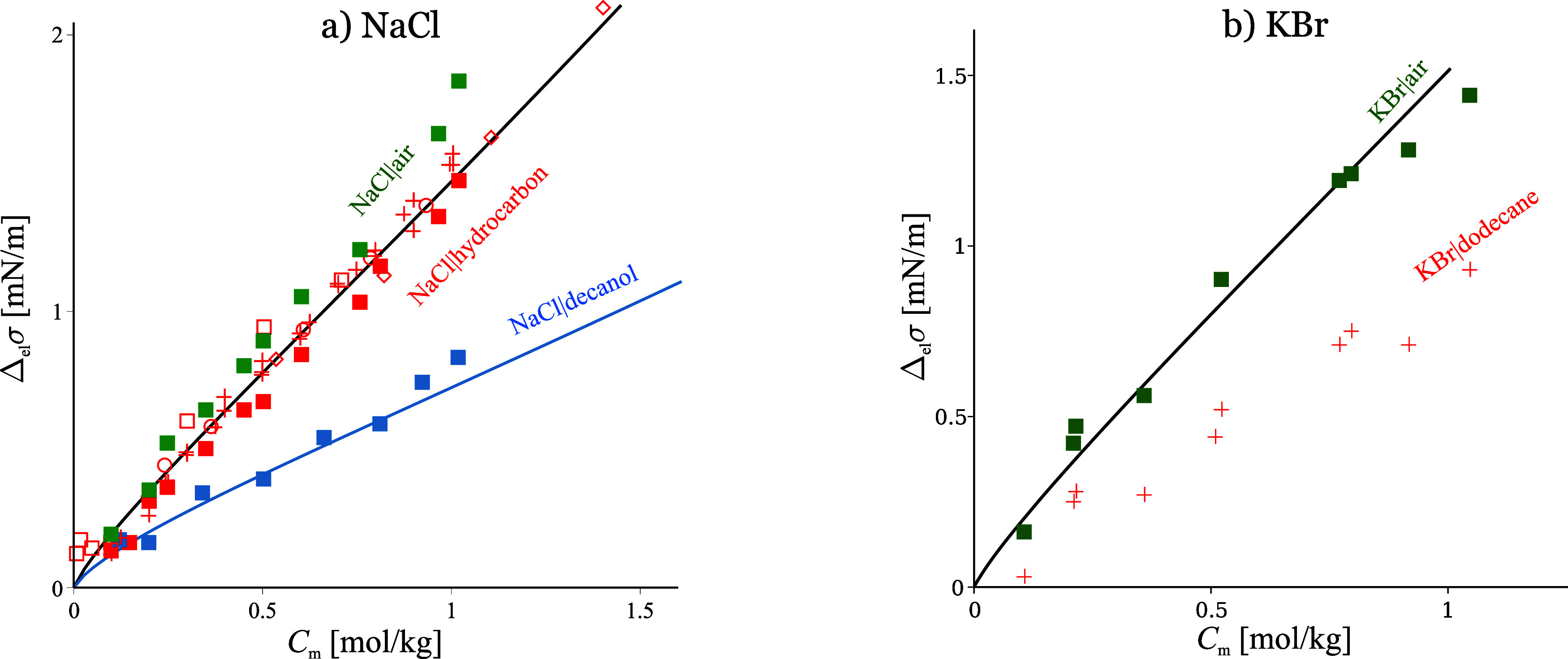
a) NaCl desorbs the same from W|A and W|H. It desorbs less from water|decanol due to the change in *z*_w_ but still follows SchM, see eq 38. Adapted with permission from ref (12). Copyright 2014 Elsevier. b) By contrast, KBr desorbs less from W|H, showing negative deviations from SchM, due to ion-dodecane dispersion interactions. Data from refs (13), (72−76) lines are SchM.

**Figure 7:**
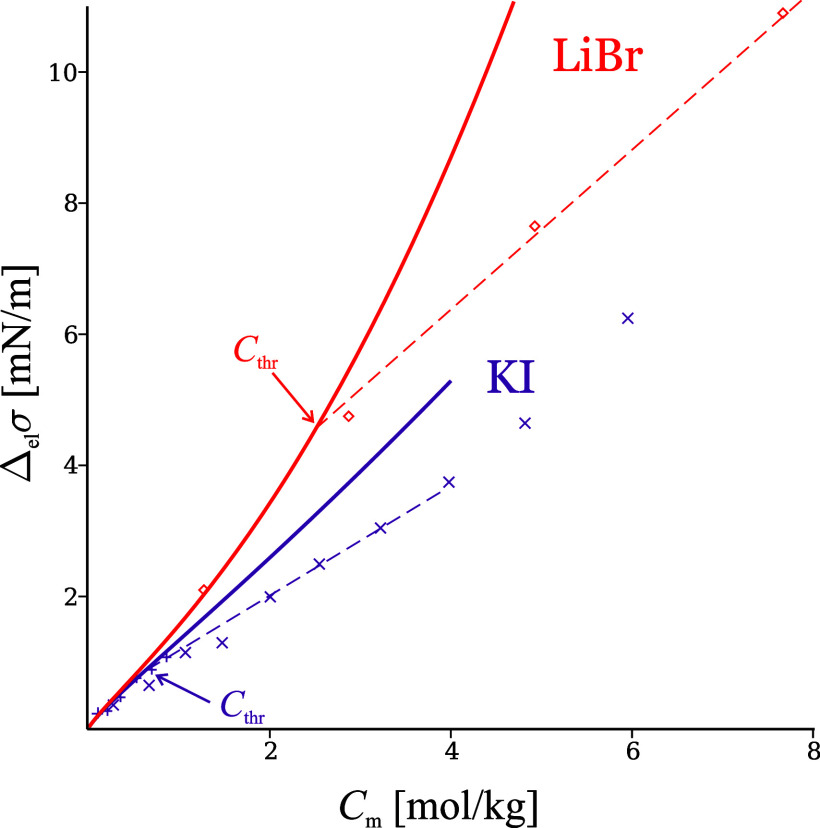
At W|A, LiBr behaves as sticky above the threshold concentration *C*_thr_ ≈ 2.5 mol/kg, and KI – only above *C*_thr_ ≈ 0.8 mol/kg. Solid lines: SchM; dashed lines: linear regression; data from refs (10), (13), and (77).

Figure 4 compares the main factors controlling *σ* of surface inactive electrolytes. The contributions to Δ_el_*σ* of the two interactions behind SchM are illustrated on the example of LiCl in Figure 4a. As seen, the image force dominates below 0.3 mol/kg. This is important when MD results are considered: MD does not handle well the long-ranged image force (i.e., the 1/*r*^4^ ion–solvent interaction). The dehydration repulsion becomes leading above 0.3 mol/kg (or 0.1 mol/kg for 2:1 electrolytes), and completely dominates the adsorption at 2 mol/kg (0.5 mol/kg for 2:1 electrolytes). For many electrolytes, the dehydration interaction alone, as defined by eqs 9–13, is sufficient to predict the surface tension from 2 to 5–10 mol/kg.

The 3-layer structure of the EDL appears to be important whenever the two ions are of very different sizes, in particular for 2:1 electrolytes at high salinity. This is illustrated in Figure 4b: if the width of the adsorption layer is ignored (i.e., the standard Gouy–Chapman model is used for the EDL), SchM predicts the “no overlap” curve that deviates from the experimental data above 2 mol/kg (NH_4_)_2_SO_4_. Since (NH_4_)_2_SO_4_ forms an adsorption layer of size comparable to that of the diffuse layer (*R*_–_ – *R*_+_ ∼ *L*_D_), the overlap effect is significant.^18^ Note that both the EDL model and the image force expression are based on the Poisson–Boltzmann equation, which is a crude approximation at high *C*_el_, and ignores effects such as ion–ion correlations in the EDL, the excluded volume, VdW interactions, quadrupolarizability of the medium etc.,^59−62^ i.e., apparently, the tensiometric data are not too sensitive to the detailed structure of the EDL. SchM agrees with the data despite these approximations because the ion–ion correlations in the bulk are accounted for through the experimental activity coefficient, while the correlations at the surface have a small effect. The Onsager image force is actually the first-order surface ion–ion correlation effect (at the Debye–Hückel level^27^) but is only significant below 0.5 mol/kg (Figure 4), while the EDL structure becomes important only above 1.5 mol/kg. The ultimate reason for the apparent validity of the Poisson–Boltzmann equation is that there are no ion correlations in a layer completely depleted from ions, and it is the depletion layer that controls the adsorption of the electrolyte; any EDL model that ensures electroneutrality will work well, as long as it takes into account the overlap effect for the calculation of the surface charge (the smaller ions expel themselves from the depletion layer of the larger ion). Even for sticky ions and for water|metal surface, the surface ion–ion correlations have been shown to be unexpectedly small, in part due to a cancelation of effects.^63,64^

Although SchM neglects all strongly specific interactions, it still predicts an ion-specific surface tension, due to two indirect ion-specific effects. The first is the ion-specific bulk density, which has a measurable effect on Δ_el_*σ* at high concentrations for a few electrolytes.^9^ The second and more important effect stems from the bulk activity coefficients *γ*_el_. It is illustrated in Figure 5: the strong ion-specificity observed for three potassium salts (*σ*_KOH_ > *σ*_KCl_ > *σ*_KNO_3__) is first of all due to their dramatically different bulk activities (*γ*_KOH_ > *γ*_KCl_ > *γ*_KNO_3__(53)). Many researchers attempt to explain the *σ* series with ion specific interactions, assuming that Γ_KOH_ < Γ_KCl_ < Γ_KNO_3__ due to stickiness of Cl^–^ and NO_3_^–^ or specific repulsion of OH^–^ while not accounting for the bulk nonideality (one early example is the apparent discrepancy between experimental and theoretical d*σ*/d*C*_el_ of 1 M KOH, KCl and KBr reported in *table 3* of ref (65)). This is incorrect: a careful differentiation according to the Gibbs isotherm (Γ_el_ = −ν^–1^d*σ*/d*γ*_el_*C*_m_ rather than – ν^–1^d*σ*/d*C*_m_) shows that the opposite adsorption series hold true: Γ_KOH_ > Γ_KCl_ > Γ_KNO_3__ (see the inset in Figure 5a), at apparent odds with the surface tension series. The same behavior is exhibited by Ca(NO_3_)_2_ and Mg(NO_3_)_2_, Figure 5b. To reiterate: the tensiometric data treated correctly show that NO_3_^–^ experiences more repulsion from the surface than Cl^–^ and OH^–^, and this conclusion is thermodynamical, valid irrespective of the model used. What SchM tells us in addition is that the source of the repulsion is the larger size of NO_3_^–^, which results in a longer-ranged dehydration force, eq 12.

Thus, for all surface inactive ions, the ion-specific values of the activity coefficient are the main reason behind the observed ion-specific surface tension. Nonideality becomes important above 0.5 mol/kg for 1:1 electrolytes (0.2 mol/kg for 1:2 electrolytes); the assumption *γ*_el_ = 1 would result in an indirect ion-specific effect being treated as direct, and, therefore, a dramatically exaggerated ion-surface attraction. This well-known effect^20,66,67,37^ has unfortunately been disregarded by several influential works in the field,^46,32,40^ which is the reason for persisting myths about the Hofmeister series at W|A. The issue appears also in the recent field theory treatment of Δ_el_*σ*,^68,69^ which corresponds to nonideal solution but at the limiting Debye–Hückel level, i.e., there ln*γ*_el_ is nonzero but is still not ion-specific.

The *surface inactive ions at W|H* also follow SchM; however, here the borderline ions Br^–^ and NO_3_^–^ behave as sticky. For example, NaCl follows SchM at both W|A and W|H, Figure 6a. By contrast, KBr follows SchM at W|A but exhibits an affinity to W|H which is not accounted for by the minimal model. Among the spectrum of expected ion-specific interactions, only the dispersion component of VdW is sensitive to whether the hydrophobic phase is air (Hamaker constant zero) or a hydrocarbon (of Hamaker constant close to that of water). Therefore, the data in Figure 6a demonstrate that NaCl at *C*_m_ < 1 mol/kg is not significantly affected by dispersion interaction at either surface. For KBr, however:

(i) there is a significant attraction of Br^–^ to W|H due to dispersion interaction with the hydrophobic phase.
(ii) Since the Hamaker constants of water and dodecane are similar, from (i) it follows that there also exists a significant dispersion attraction of Br^–^ toward the aqueous phase, which should result in repulsion from W|A.
(iii) Since KBr follows closely SchM at W|A, the dispersion repulsion from (ii) must be compensated by an attractive force that acts at both W|A and W|H; most likely, this is a combination of hydrophobic interaction with Br^–^ and the Debye ion–water force.

We can therefore conclude that the validity of SchM for borderline-sticky ions like nitrate and bromide at W|A to such high concentrations is the fortuitous result of a cancelation of effects: London repulsion and hydrophobic and Debye attraction. Since the London force is longer-ranged, the same forces will produce sizable electric polarization (see the Supporting Information). Moreover, these interactions are of much smaller magnitude for “proper” surface inactive ions.

For sticky ions, the hydrophobic attraction dominates over VdW; in fact, at W|H (absent dispersion repulsion), the hydrophobic force acting on, e.g., I^–^ is so strong that it wins over even the dehydration and image force^12^ – the iodides LiI, NaI and KI have positive adsorption (the interfacial tension *σ*^W|H^ drops with *C*_el_). At W|A, where the dispersion repulsion is added to the force mix, the total surface force acting on the same iodides produces a mild (compared to SchM) desorption, see Figure 7. Variants of this picture are incorporated in the theories of Levin et al. and Ninham et al.^46,40^ (even if the two effects are exaggerated for surface inactive ions) but not, e.g., in our old work on ion-specific effects at charged monolayers.^5,6^

Another test that allows to investigate the contribution of the VdW force is the comparison of the adsorption of ions of similar size but different polarizability. Such ions should exhibit the same image, dehydration and hydrophobic but different VdW interactions. Several examples of such situation are

(i) NaNO_3_ follows closely SchM while AgNO_3_ exhibits^9−11^ positive deviations from the model corresponding to additional repulsion (the cationic radii are close while polarizabilities differ – *α*_*i*_/4πε_0_ = 0.26 Å^3^ for Na^+^ vs 2.0 Å^3^ for Ag^+^);
(ii) K_2_SO_4_ follows SchM, but K_2_CrO_4_ shows deviations in direction of repulsion^12,10^ (polarizabilities 5.5 Å^3^ for SO_4_^2–^ vs 10.5 Å^3^ for CrO_4_^2–^);
(iii) CaCl_2_ follows SchM, while CdCl_2_^12,10^ exhibits additional repulsion (polarizabilities 0.63 Å^3^ for Ca^2+^ vs 1.28 Å^3^ for Cd^2+^). The data for CdCl_2_ is complicated by expected complexation of this salt in the solution; however, the complexes are expected to adsorb more than the free ions, so the repulsion must be stronger than just *u*_im_ + *u*_h_.

In all three cases, the effect is fairly mild: at most 20% deviation of Δ_el_*σ* from SchM at high concentration.

#### Critical Penetration Concentration of Sticky Ions into the Depletion Layer

Electrolytes containing monovalent ions larger than 1.95–2.0 Å show small negative deviations from SchM at W|A. This is clear evidence that ClO_3_^–^, HCOO^–^, SCN^–^, I^–^, ClO_4_^–^ are attracted to the water surface. The borderline size is slightly larger than the largest cavity, 1.6 Å, in hexagonal ice, which is a useful approximation for the structure of liquid water; therefore, the threshold appears to be related to the hydrophobic effect (larger ions disrupting the hydrogen bond net in water).^9^

The borderline between sticky and surface inactive ions is somewhat dependent on the case studied: stickiness is more pronounced at W|H; the surface potential is more sensitive to stickiness than surface tension; and importantly, the stickiness is concentration-dependent.^9^ The comparison with SchM reveals that the attraction to W|A affects *σ* only above a certain threshold concentration, as if the depletion layer leaches the ions only after a certain activation chemical potential of the electrolyte is surpassed. Moreover, it turns out that borderline ions (Br^–^, NO_3_^–^) also exhibit such behavior, only the threshold appears at a very high concentration.

Two examples are shown in Figure 7. The threshold concentration *C*_thr_ is often difficult to determine accurately and it is not possible to say how sharp the transition is, due to experimental uncertainty and lack of sufficient data in the region where the change takes place; nevertheless, Table 1 presents an approximate (±0.5 mol/kg) summary of the data collected in the supplement of refs (9) and (18). The upper right corner of this table corresponds to electrolytes of wide nonsticky region (e.g., NaCl follows SchM up to its solubility limit); the lower left corner corresponds to very sticky electrolytes which adsorb more than SchM predicts even at the lowest reported concentration.

**Table 1:** Upper Concentration Limit of Validity of SchM, *C*_thr_ [mol/kg], for Various Electrolytes^a^

	NH_4_^+^	Li^+^	K^+^	Na^+^
Cl^–^	*C*_thr_ ≈ 3	3	>solubility limit	>solubility limit
Br^–^	1	2.5	3	5–6
I^–^	<0.3	<0.3	0.8	0.8
HCOO^–^	-	<0.3	<0.4	0.8
ClO_3_^–^	-	-	<0.1	0.7

a Values are based on data from ref (9) and determined as in Figure 7.

There is a clear regularity in the values of *C*_thr_ (unnoticed previously in refs (9) and (18)) – the ion order followed by *C*_thr_ (from highest *C*_thr_/least sticky to lowest *C*_thr_/most sticky) is
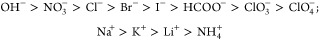
21

This is the closest one can get to an order of the affinity of ions to W|A, obviously quite different from Hofmeister’s series (1) and (2). The anion stickiness, as measured by *C*_thr_, correlates positively with the ion radius (radii of OH^–^ < NO_3_^–^ ≈ Cl^–^ < Br^–^ < ClO_3_^–^ < HCOO^–^ < I^–^ < ClO_4_^–^). HCOO^–^ and ClO_3_^–^ appear to be exceptions from this correlation: these two ions have a significant dipole moment which should contribute to the repulsive dipole image force, making them less sticky. The cation series also correlates positively with the ion radius, except for Li^+^.

For the large anions, the reason for this threshold behavior may be, by analogy with the theory of the EDL at water|mercury,^38^ that the ion has to lose a part of its hydration shell in order to penetrate into the “inner Helmholtz layer”, i.e., to approach the physical surface to a distance equal to the bare ion radius. For example, for iodide, this corresponds to the process I(H_2_O)_n_^–^ ⇄ I(H_2_O)_m_^–^ + (n–m)H_2_O, where associates I(H_2_O)_m_^–^ of smaller hydration number m adsorb more. Another possible reason for the appearance of *C*_thr_ is the formation of ion pairs. This is expected for lithium, magnesium and calcium salts (Mg(NO_3_)_2_ and Ca(NO_3_)_2_ are of *C*_thr_ ≈ 1.5 and 3 mol/kg, respectively^18^). The LiBr dependence in Figure 7 can be reproduced with assumed dissociation equilibrium LiBr ⇄ Li^+^ + Br^–^ of equilibrium constant of the order of 20–30 mol/kg, and ion pair LiBr of zero or slightly positive adsorption. To explain why LiCl is less surface active than LiBr (while being more prone to forming ion pairs^53^), one must further assume that the respective ion pairs have different adsorption constants. Similar effect should be expected in cases where *complexation* of ions takes place; cations of d- and f-elements tend to speciate with their counterion differently in the bulk and at the surface.^78^ Partial hydrolysis will have a speciation effect of another kind, important for ions like H_2_PO_4_^–^, which can disproportionate to HPO_4_^2–^ and surface active H_3_PO_4_; hydrolysis is also essential for the understanding of the pH dependence of the electrolyte adsorption.^11^ Such association phenomena were thought to be the main reason for surface ion specificity by Langmuir.^79^

The break in the experimental *σ*(*C*_el_) corresponds to an abrupt change of the derivative d*σ*/d*C*_el_, adding complexity to the Marangoni effect in thinning foam films (one should expect nonmonotonous concentration dependence of the theoretical^56^ coalescence time). It also limits the validity of the interpretation^57^ of the αβ rule of Craig et al.^54^ based on the value of d*σ*/d*C*_el_ at neat W|A. One example for this is NaClO_3_, an αβ electrolyte having no effect on coalescence^54^ – this salt behaves as sticky only above 0.7 mol/kg, while in the concentration range studied by Craig et al., it follows SchM accurately,^9^ with value of d*σ*/d*C*_el_ similar to that of any αα electrolyte.

The *permanent dipole moment of the ion* is clearly a factor. We already mentioned that HCOO^–^ and ClO_3_^–^ violate the stickiness vs ion size correlation in eq 21, as if they are less sticky than their radii would suggest. Similarly, hydroxides show additional weak repulsion compared to SchM (NaOH, LiOH, Ba(OH)_2_), and so do NaBrO_3_ and NaIO_3_.^9,18^ The repulsion is the expected result of the Keesom and Debye interactions with the aqueous medium of the dipole carried by the ion.

#### Wagner’s Effect

When Wagner proposed the expression for the screened image potential (7), he attempted to introduce an increased surface Debye length producing weaker screening due to depletion of ions. Onsager and Samaras^27^ showed that Wagner’s approach is not entirely consistent and neglected the effect. Based on the deviations of the data from the SchM, this seems reasonable for 1:1 and 2:1 electrolytes. However, all 2:2, 3:1 and 3:2 electrolytes show significant positive deviations from SchM (e.g., Cr(NO_3_)_3_ in Figure 5b). Wagner’s effect corresponds to a second order correlation effect (Onsager-Samaras setting the first order), or equivalently, to an excess of surface conductivity. The surface conductivity is known to affect the image force,^80^ although here we speak of a negative surface conductivity, which produces a singularity in the available theoretical expressions. The field theory of Markovich et al.^68,69^ should allow a consistent treatment of the effect, but, to our knowledge, it has not been applied to multivalent electrolytes yet.

*Hydrogen bonding* has been identified^81^ as the reason why H_3_O^+^ behaves as a sticky ion.^82,83^ An explanation other than hydrogen bonding is difficult from the viewpoint of finding cognate ions of similar behavior: e.g., ions of similar size (1.3 Å^34^), polarizability, and dipole moment do not show affinity to W|A. On the other hand, the leading place of NH_4_^+^ in the *C*_thr_ series (21) and the big difference in *C*_thr_ of ammonium and potassium salts (despite the similar size of the two ions) suggest that NH_4_^+^ has a similar in nature, but weaker, stabilizing effect on the hydrogen bond network at the surface (confirmed also by sum frequency generation spectra^84^). Moreover, the neutral NH_3_, which is isoelectronic with H_3_O^+^, has a comparable affinity to W|A^85^ – the derivative d*σ*/d*C* of NH_3_ is similar to the differences d*σ*/d*C*(HCl) – d*σ*/d*C*(KCl) or d*σ*/d*C*(HNO_3_) – d*σ*/d*C*(KNO_3_) (based on data from ref (10)), which are a measure of the affinity of H_3_O^+^ to W|A compared to a surface inactive cation.

The deviations from SchM are often positive at low concentrations but become negative at higher. One example is OH^–^, which experiences extra repulsion from the surface compared to SchM due to the image force acting on its permanent dipole, but, at very high concentrations where this image force is screened, there is evidence of attraction (*C*_thr_ ≈ 5.5 mol/kg for KOH), likely due to hydrogen bonding similar to H_3_O^+^ but weaker. MgSO_4_ is showing strong Wagner’s effect below 3 mol/kg, but the tensiometric data approach the SchM in more concentrated solutions, due to screening of the image force, and also because the free water (not included in the hydration shells of the ions) in 3 mol/kg MgSO_4_ is depleted, and a population of dehydrated ions and ion pairs exists in this solution, resulting in weaker desorption.

Let us here discuss briefly the nonaqueous electrolyte solutions, which have recently attracted significant research interest.^86−89^ In organic solvent of dielectric permittivity lower than water, electrostatic forces are stronger, i.e., image force is more repulsive (1/*ε* in eq 7) and the bulk solution is more nonideal. The desolvation force should become longer-ranged and less ion-specific due to the larger solvent size − *R*_w_ in eq 12 is substituted by a larger *R*_solvent_. Of the specific forces, the hydrophobic attraction (dominated by proton disorder) must decrease. Therefore, it can be expected that the ion-specific effects on *σ* will be even more dependent on indirect effects, in particular on ion association in the bulk. The detailed test of this expectations requires data for *γ*_el_, but the diminished hydrophobic attraction for I^–^ seems to be confirmed by the tensiometric data in formamide and glycerine.^88,89^ Moreover, in formamide (more polar and bulkier than water), the gradient d*σ*/d*C*_m_ at 1 mol/kg of a range of alkali halogenides is indeed larger than for aqueous solution, as expected from the longer-ranged desolvation force, and is not very ion-specific.^88^ For glycerol (less polar than water), the alkali halogenides are of gradient d*σ*/d*C*_m_ lower than the aqueous solutions and the value of *σ* varies with the ion, which is probably an indirect ion-specific effect caused by ion pairing in the bulk, producing a strongly negative ln*γ*_el_ and relatively surface-active ion pairs.

### Surface Potentials: EDL and Dipolar

The modified Schmutzer model predicts also the potential *ϕ*^DL^ of the EDL^18^ (i.e., the potential drop through the surface due to the distribution of ions, including the response of the solvent through *ε* but excluding the intrinsic dipole moment of the surface). Similarly to the adsorption (14), *ϕ*^DL^ can be shown to be separable to two addends, *ϕ*^DL^ = *ϕ*_h_^DL^+ *ϕ*_im_^DL^. The reported data for surface Δ*χ* potentials is at ionic strength above 200 mM^90,91,15^ where the image force contribution *ϕ*_im_^DL^ is negligible; therefore, *ϕ*^DL^ is dominated by the dehydration potential. The following expression was derived for it:^18^
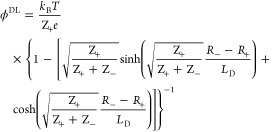
22this result is valid for *R*_–_ > *R*_+_; in the opposite case, the indices “+” and “–” must be exchanged, and *ϕ*^DL^ changes sign (a minus appears in front). The potential predicted by eq 22 is of the order of 5 mV for 1:1 electrolytes, and up to 20 mV for 2:1 salts. Most 1:1 electrolytes have a small cation standing closer to the surface than a larger anion; therefore, *ϕ*^DL^ predicted by SchM is positive toward the air.

By contrast, the measured Δ*χ* potential is usually negative for 1:1 electrolytes. The negative experimental Δ*χ* is one of the reasons why the general opinion in the literature is that, e.g., aqueous NaCl is of negative *ϕ*^DL^(39,49,90) (although the opposite claim has also been made^69^). To explain the negative Δ*χ*, Randles,^90^ among others, assumed that Δ*χ* ≈ *ϕ*^DL^ and postulated an attractive potential acting on Cl^–^ to produce *ϕ*^DL^< 0. However, a direct test of the effect of such hypothetical potential on the adsorption of chlorides at W|A shows that it also produces a significant adsorption and a decrease in surface tension, in disagreement with the tensiometric data.

The surface Δ*χ* potential is, in fact, the sum of *ϕ*^DL^ and the change in the dipolar potential, as highlighted by Frumkin^91^ and Overbeek.^92^ The assumption Δ*χ* ≈ *ϕ*^DL^ requires that the surface of a concentrated electrolyte carries the same nonionic normal dipole moment as pure water. This is, of course, never quite true; Frumkin, for example, was careful not to equate Δ*χ* to *ϕ*^DL^ whenever |Δ*χ*| is less than 5–10 mV, and there is one known example where Δ*χ* and *ϕ*^DL^ are of different signs: HCl has negative Δ*χ*^91^ while H^+^ is clearly sticky^82^ and is charging all hydrophobic surfaces positively.

To estimate the effect of the electrolyte on the dipolar component of Δ*χ*, a model is needed for a structure existing at the surface called the *dipolar double layer*.^23^ In short, the interface between pure water and a hydrophobic phase is polarized due to specifically adsorbed normal dipole *P*, caused probably by the cross dipole-quadrupole image force acting on water.^93−95^ This surface dipole creates an electric field conjugated with the bulk quadrupolarization, that extends to around an ångstom (1–2 quadrupolar lengths *L*_*Q*_) away from the location of the adsorbed surface dipole. The electric field orientates the surrounding solvent molecules in the opposite direction of *P*, thus creating an oppositely polarized diffuse dipolar layer.^23^ The total potential drop is the result of the combined contributions of the adsorbed and the diffuse dipolar layers, plus the EDL potential:
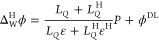
23Here, *L*_*Q*_ is the quadrupolar length of water, *L*_*Q*_^H^ is the quadrupolar length of the hydrophobic phase, and *ε*^H^ is its dielectric permittivity. The quadrupolar length is a ratio between the quadrupolar and the dipolar strength of the medium^23^ and sets the thickness of the dipolar double layer (similarly, the Debye length is a ratio between dipolar strength and ionic strength and sets the thickness of the EDL). *L*_*Q*_ varies from ∼0.5 Å for polar solvents up to ∼1.5 Å for nonpolar strongly quadrupolar fluids such as benzene and liquid CO_2_.^96,97^ For W|A, *L*_*Q*_^H^ = 0, so eq 23 simplifies:
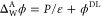
24

The adsorbed layer of dipoles alone produces a potential drop *P*/ε_0_, but this is nearly exactly compensated by the diffuse layer of dipoles, resulting in potential of only *P*/*ε*, which is 2 orders of magnitude lower than *P*/ε_0_.^23^ In comparison, the classical Helmholtz condenser model would assume dipolar contribution *P*/*ε*^S^ to Δ*χ*, where *ε*^S^ is a hypothetical local dielectric constant of the medium in the adsorbed layer of dipoles (cf. refs (98) and (99)); eqs 23 and 24 show that, for a thin adsorption layer, the dielectric response is essentially nonlocal, and it is the ion-specific bulk permittivity *ε* that controls the medium response to the field created by *P*.

The Δ*χ* potential is the difference of the surface potentials Δ_W_^A^*ϕ*_el_ of the electrolyte solution and Δ_W_^A^*ϕ*_w_ of pure water
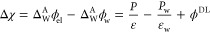
25where Δ_W_^A^*ϕ*_w_ = *P*_w_/*ε*_w_ (pure water’s adsorbed dipole *P*_w_ over pure water’s dielectric permittivity). The adsorbed normal dipole moment *P* is due to the water molecules in the surface layer, of surface density Γ_p_, each molecule carrying an average dipole of *p*:
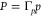
26

Therefore, the electrolyte can alter the dipolar contribution to Δ*χ* via the following effects:

(i) by reducing the bulk dielectric permittivity *ε* compared to *ε*_w_. This phenomenon can be dealt with directly, by using literature data for *ε*(*C*_m_) of the electrolyte solution.^60,100^ It is a strong effect: *ε*_NaCl_/ε_0_ drops to 45 at 5 mol/kg,^100^ so if pure water has Δ_W_^A^*ϕ*_w_ = *P*/*ε*_w_ = −100 mV,^101,102^ the dipolar contribution to Δ_W_^A^*ϕ*_el_ will drop to −175 mV solely due to the decrease in *ε*, producing a –75 mV contribution to Δ*χ*.
(ii) Alteration of Δ*χ* through a change in the average dipole moment *p*. This effect requires an investigation of the interaction between the electric double layer and the dipolar double layer, and also a model of the polarization of the aqueous surface by, e.g., the quadrupole image force acting on water; both are currently missing. A recent investigation into the question^95^ suggests that *p* should decrease with the addition of electrolyte, but, in the absence of a developed theory, we assume *p* = *const*.
(iii) Alteration of the number Γ_*p*_ of water dipoles contributing to *P*. The number of water molecules in the top layer of the neat surface is

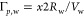
27where *x* is a fraction of value of the order of 0.5, judging from the value of the thickness of the hydrophobic gap. If an ion has its hydration shell occupying a fraction of the depletion layer (see Figure 2), and the water in the shell is fully orientated, then the value of Γ_*p*_ will be reduced by *N*_w_ water molecules per ion in the subsurface layer, corresponding to
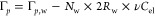
28

By combining eqs 25−28, we arrive at the following expression for Δ*χ*:^18^
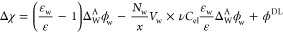
29

We previously tried to determine the two unknown parameters in eq 29, *N*_w_/*x* and Δ_W_^A^*ϕ*_w_, via regression over the experimental Δ*χ* data.^18^ However, eq 29 is only a rough approximation; moreover, the experimental data for both Δ*χ* and *ε* are very uncertain. Therefore, we ended up using *N*_w_/*x* = 3 (based on the geometry of the hydration shell) and Δ_W_^A^*ϕ*_w_ = −100 mV^101^ as rough estimates, and to focus on the qualitative features of the results. The formula (eq 29) can be corrected for the ion depletion at the surface (as suggested by Marcus^33^): the image potential (7) modifies *C*_el_ in eq 28 with a Boltzmann factor exp[−*u*_im,*i*_(*z*)/*k*_B_*T*], where *z* is in the range *R*_*i*_...*R*_*i*_+2*R*_w_ (the respective EDL potential contributions to the subsurface concentration of the cation and the anion cancel). Substituting ν*C*_el_ for Σν_*i*_*C*_el_exp(−*u*_im,*i*_/*k*_B_*T*) in eq 28 is equivalent to a 35% correction in the empirical number *N*_w_ for salts made of small ions at 1 M. This correction improves the agreement between the model and the experimental Δ*χ* data for NaCl and LiCl, which are most affected; however, *N*_w_/*x* itself is not known (our guess *N*_w_/*x* = 3 cannot claim precision better than ±50%); moreover, the uncertainty in the value of Δ_W_^A^*ϕ*_w_ and the approximation *p* = *const* are worse sources of error; therefore, we neglect the Boltzmann factor correction for simplicity.

**Figure 8:**
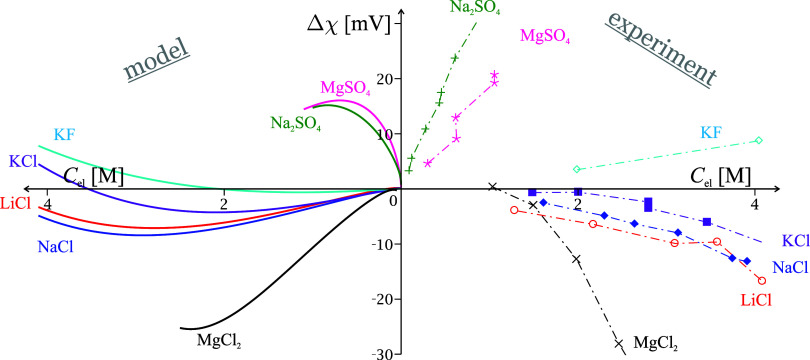
Surface Δ*χ* potential of solutions of surface inactive electrolytes. On the left: eqs 29 and 22, combining SchM for the EDL potential and the theory for the effect of the electrolyte on the dipolar contribution to Δχ. On the right: experimental potentials from Kenrick’s jet method^91^ and radioactive probe measurements.^15^ Two equivalent positive *C*_el_-axes are used on the two sides of the Δ*χ*-axis. Adapted with permission from ref (18). Copyright 2013 Elsevier.

For *symmetric electrolytes and surface inactive ions*, the dipolar contribution to Δ*χ* dominates, and the ion-specificity of *ε* appears to be the reason behind the ion-specificity of Δ*χ*. This is illustrated in Figure 8: the model (29) correctly predicts that Δ*χ*_LiCl_ ≈ Δ*χ*_NaCl_ < Δ*χ*_KCl_ < Δ*χ*_KF_, and this order follows exactly *ε*_LiCl_ ≈ *ε*_NaCl_ < *ε*_KCl_ < *ε*_KF_. Moreover, the reason for the negative sign of Δ*χ* of 1:1 surface inactive salts is, first of all, the decrement of *ε* with the concentration of electrolyte (the first term in eq 29). For KF, this decrement is weak, and the effect from the reduction of Γ_*p*_ (second term in eq 29) starts to dominate, producing a positive Δ*χ*. One particularly interesting case is MgSO_4_: this symmetric electrolyte has the unique feature of elevating,^60^ rather than decreasing, the dielectric permittivity of the solution. This explains its large positive Δ*χ*.

For *2:1 surface inactive electrolytes*, like Na_2_SO_4_ and MgCl_2_, Δ*χ* is dominated by the EDL charging due to the disparity in hydration (last term in eq 29 given by eq 22) – the small ions Na^+^ and Cl^–^ charge the adsorption layer while the strongly hydrated Mg^2+^ and SO_4_^2–^ remain in the diffuse layer. This behavior is captured well by our minimal model (Figure 8).

With most *sticky ions*, Δ*χ* is clearly affected by the EDL contribution *ϕ*^DL^. This includes the borderline ions Br^–^ and NO_3_^–^: 2 mol/kg NaBr and NaNO_3_ have Δ*χ* ∼ – 15 mV^15^ which is difficult to explain with a change of the dipolar contribution alone. The charging becomes observable below *C*_thr_; thus, the surface potential is more sensitive to the interactions behind the ion stickiness compared to the adsorption and surface tension. This sensitivity is in line with the conclusion we made about Br^–^ and NO_3_^–^ from the tensiometric data, for longer-ranged VdW repulsion and shorter-ranged hydrophobic attraction: even if the two contributions cancel in the adsorption at W|A, the difference in range of action will still produce polarization and a respective negative ionic potential that contributes to Δ*χ* – a case discussed in the Supporting Information. This concentration polarization explains why Br^–^ adsorbs less than Cl^–^ yet bromides produce more negative Δ*χ* potentials compared to chlorides. This picture is confirmed also by MD^49^ (though somewhat exaggerated in comparison with the data).

With larger ions, the Δ*χ* potential becomes large negative (e.g., 2 mol/kg KI and NaI approach −50 mV). However, the assumption Δ*χ* = *ϕ*^DL^ is even worse for such ions–if I^–^ populates the surface layer, then it certainly has a large effect on the dipolar component of Δ*χ*, through replacing polarized water from there (eq 28 but with a larger *N*_w_). Moreover, the partial dehydration required for the sticky ion to penetrate the depletion layer produces a permanent dipole of the hydrated ion itself, which contributes directly to *P*. This is why the EDL potentials calculated by Levin and Dos Santos^40^ are low compared to Δ*χ* data for sticky ions at 1 mol/kg (even though their model neglects the repulsive dispersion and is, therefore, exaggerating *ϕ*^DL^).

Ions that carry an intrinsic dipole moment (H_3_O^+^, OH^–^, HCOO^–^) will also contribute to the surface dipole moment *P* directly, via orientation by the Debye image ion-dipole interaction: anion–dipoles will point with their positive pole toward the surface, i.e., dipolar anions should produce more positive Δ*χ* potential; on the opposite, dipolar cations produce more negative potential. The example here is H_3_O^+^, where *ϕ*^DL^ is strongly positive, while Δ*χ* is strongly negative. The effect is, of course, more complicated than that, because acids are likely to decrease *ε* more strongly than other electrolytes (the proton is nonpolarizable and, moreover, it produces strong dielectric saturation), so the main effect may be a particularly strong drop in *ε*.

NaOOCCH_3_ is an example where the adsorbed species is orientated not only by the Debye force but also by the hydrophobic potential; the outcome is a large positive Δ*χ*,^15^ i.e., the dipole contribution from the adsorbed CH_3_COO^–^ (positive pole toward the air) wins over the negative *ϕ*^DL^.

At the end of this section, it should be mentioned that water’s normal surface dipole moment *P* interacts with ions. The water molecules point with the oxygen end toward air; therefore, the ion-surface dipole interaction attracts anions to the surface and repulse cations.^103^ The total contribution to the adsorption nearly cancels, but this interaction polarizes the surface by a few mV through *ϕ*^DL^. The satisfactory description of this effect is still lacking, because the interaction is complex and requires the equations of quadrupolar electrostatics to be used, including an accurate equation of state for the normal dipole moment vs the surface electric field.^23,42^

## Ion Adsorption on Uncharged Monolayers

### Theory of Electrolyte Adsorption on Uncharged Monolayers

The adsorption of electrolyte on a surfactant monolayer is not normally directly accessible via tensiometry. The problem is that the Gibbs isotherm contains the chemical potential of both the electrolyte and the surfactant:
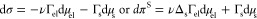
30Here, *π*^S^ = *σ*_0_ – *σ* is the surface pressure of the monolayer; Δ_s_Γ_el_ = Γ_el_ – Γ_el,0_ is the *monolayer-induced electrolyte adsorption*, a somewhat more direct characteristic of the ion-monolayer interaction than Γ_el_ alone;^17^*π*^S^ and Δ_s_Γ_el_ are thermodynamic conjugates (while Γ_el_ is coupled with *σ*); *σ*_0_ and Γ_el,0_ are surface tension and electrolyte adsorption of the surfactant-free surface. In the absence of surfactant (Γ_s_ = 0), the dependence of *σ* on *C*_el_ can be converted directly to adsorption through the derivative Γ_el_ = −ν^–1^d*σ*/d*μ*_el_. By contrast, in the presence of a soluble surfactant of concentration *C*_s_ in the aqueous solution, if the concentration of the electrolyte is varied, the Gibbs isotherm gives for Γ_el_:
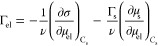
31i.e., to find Γ_el_, one needs independent information about how the electrolyte salts out the surfactant to calculate the derivative (∂*μ*_s_/∂*μ*_el_)_*C*_s__.^12^ The same issue arises with a spread monolayer of insoluble surfactant, where the experimental data for *σ* vs *C*_el_ at constant area per molecule is insufficient to determine Γ_el_ in the absence of knowledge of the derivative (∂*μ*_s_/∂*μ*_el_)_Γ_s__, measuring the effect of the salt in the substrate on the free energy of a monolayer of fixed density.

There are a few exceptional cases where *σ* vs *C*_el_ can be determined at fixed chemical potential *μ*_s_ of the surfactant. One option is adsorbing an oil-soluble surfactant at the electrolyte solution|oil interface. In this case, the oil solution controls *μ*_s_ with no interference by the electrolyte in the water and ∂*μ*_s_/∂*μ*_el_ = 0. This allowed Aveyard et al.^73^ to determine the salt adsorption Γ_el_ on such monolayers. Another system where *μ*_s_ is maintained constant is the equilibrium spread monolayer around a crystal or a droplet of the surfactant phase. Provided that the surfactant phase dissolves neither electrolyte nor water, it again acts as a container keeping the chemical potential of the equilibrium spread monolayer equal to *μ*_s_ = *μ*_s_^°^ of the surfactant phase. The adsorption Γ_el_^°^ of various electrolytes on such monolayers has been studied via the surface pressure *π*° of the equilibrium spread monolayer;^104−108^ the derivative d*π*°/d*μ*_el_ gives the monolayer-induced electrolyte adsorption on the equilibrium spread monolayer:
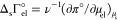
32

The electrolyte adsorption on insoluble monolayers is actually accessible tensiometrically but through a combination of experiments. The idea was proposed by Frumkin and Pankratov^105^ but was realized only recently by us.^102,17,109^ The approach requires simultaneous determination of spreading pressure *π*° of the surfactant phase and standard compression isotherms *π*^S^ vs Γ_s_ at several electrolyte concentrations. From these data and eq 30, one can calculate the molar Gibbs energy for producing a monolayer from a crystal:
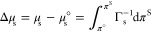
33

Δ*μ*_s_ is the chemical potential of the monolayer with respect to an electrolyte-independent reference state, namely the surfactant phase; the dependence of Δ*μ*_s_ on *C*_el_ at constant Γ_s_ carries information about how the electrolyte in the substrate stabilizes or destabilizes the monolayer^12,17^ and is informative on its own. Once Δ*μ*_s_ is known, the electrolyte adsorption follows either from the Gibbs–Duhem relation between Δ_s_Γ_el_ and Γ_s_^105,102^ or from ∂*π*^S^/∂μ_el_ at fixed Δ*μ*_s_:^17^
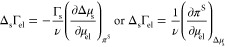
34

The comparison of the two routes to find Γ_el_ allows one to test the data for thermodynamic compatibility and provides an idea about the uncertainty through the difference between the two derivatives in eq 34.

Let us now discuss how the presence of adsorbed surfactant affects the location of the physical, dielectric and equimolecular surfaces. The polar head groups of the surfactant occupy a large fraction of the volume of the surface layer; therefore, eq 17 has to be modified to accommodate this volume:^12,17^
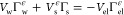
35

*V*_s_^*ε*^ stands for the volume of the surface layer below the dielectric surface occupied by the surfactant functional group. A strongly polar headgroup (R–OH, R–COOH) will penetrate the surface layer without reducing its polarity, hence, *V*_s_^*ε*^ is approximately equal to the volume of the headgroup *V*_head_ in this case. For weakly polar head groups (R–OC_2_H_5_, R–Br), the protrusion into the top layer is simultaneously shifting the location of the dielectric surface toward the aqueous solution, and the outcome is that *V*_s_^*ε*^ ≪ *V*_head_. One approximate estimate of *V*_s_^*ε*^ is
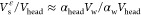
36Here, *α*_*i*_ is the contribution of component *i* to the macroscopic polarizability of a fluid. For example, the —OH group of alkanols is expected to substitute one molecule of water from the top layer (*V*_head_ ≈ *V*_w_) without changing the dielectric profile (*α*_head_ ≈ *α*_w_), which means that for alcohols *V*_s_^*ε*^ ≈ *V*_w_.^12,102^ For a carboxylic acid, however, the weakly polar >CO group cannot be treated the same way as the —OH moiety.^17^ The polarizability of —COOH can be estimated as *α*_*i*_ = (*ε*_*i*_ – ε_0_)*V*_*i*_, where *ε*_*i*_ is the dielectric permittivity of a liquid that approximates the moiety *i* (e.g., HCOOH). Using the values *V*_w_ = 18 mL/mol, *V*_head_ ≈ *V*_HCOOH_ = 38 mL/mol and *ε*_HCOOH_/ε_0_ = 51 (where HCOOH indicates the properties of liquid formic acid; a small correction for the contribution *α*_tail_ is made assuming 2–3 CH_2_ groups contributing to *V*_s_^*ε*^), we find that *V*_head_^*ε*^ ≈ 28 mL/mol, i.e., the effective dielectric volume *V*_s_^*ε*^ of the —COOH group is between that of the actual volume of —COOH, 38 mL/mol, and the volume of just the —OH group, 18 mL/mol.

From eq 35, the adsorption of water at the dielectric surface follows, together with the corresponding location *z*_w_ of the equimolecular surface:
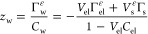
37

This equation generalizes eq 18 to a surface covered with surfactant. For a dense monolayer, the term *V*_s_^*ε*^Γ_s_ dominates in eq 37: the shift of the equimolecular surface with respect to the dielectric one upon adsorption of a monolayer is approximately *z*_w_ = −*V*_s_^*ε*^Γ_s_. Effectively, the surfactant polar groups are pushing the equimolecular surface of water toward the aqueous solution and the ions, which corresponds to an effective adsorption of electrolyte. This is reflected by the Gibbs adsorption of the electrolyte at *z* = *z*_w_:
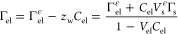
38

This result generalizes eq 19. The extra electrolyte adsorption *z*_w_*C*_el_ ≈ – *V*_s_^*ε*^Γ_s_*C*_el_ can be treated as an osmotic effect caused by the polar head groups diluting the surface layer.^12^

Thus, our minimal model predicts that even if the adsorption Γ_el_^*ε*^ of electrolyte at the dielectric surface is the same for surfactant-free W|A and for water|monolayer, the Gibbs adsorption Γ_el_ is nevertheless going to increase at water|monolayer, due to the osmotic effect on *z*_w_*C*_el_. The effect can be demonstrated by comparing the adsorption of electrolyte at W|H and water|decanol phase. At water|decanol, the number Γ_s_ of hydroxyl group protruding the surface layer corresponds approximately to a packed monolayer, 1/Γ_s_ = 18.2 Å^2^. Substitution of eq 38 for Γ_el_ under the integral (20), with Γ_el_^*ε*^ given by SchM, indeed^17^ produces a prediction in agreement with the available data for LiCl, KCl and NaCl at water|decanol (Figure 6).

We can combine eqs 19 and 38 to calculate the monolayer-induced electrolyte adsorption:
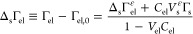
39

For small surfactant head groups, SchM predicts the same adsorption Γ_el_^*ε*^ on the dielectric surface with and without the surfactant monolayer, so Δ_s_Γ_el_^*ε*^ ≈ 0. This means that the SchM “baseline” for Δ_s_Γ_el_ is given by a linear osmotic adsorption isotherm:
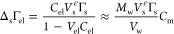
40

Deviations from this baseline indicate the magnitude and the direction of any specific ion-monolayer interaction, through an analysis similar to that of neat surfaces data in sec. *Electrolytes at W|A and W|H: interfacial tension*. By contrast, for bulky surfactant head groups, the range of the “desolvation” force also changes: an ion solvated by the headgroup has a new *R*_h,*i*_, involving the size of the headgroup rather than *R*_w_ in eq 11; moreover, the density of the surface layer changes,^110^ inducing a respective change in *z*_h_. Therefore, Δ_s_Γ_el_^*ε*^ is not generally zero. In any case, the substitution of water in the hydration shell with surfactant headgroup is introducing a specific interaction which is beyond SchM.

The case of salt interacting with nonionic surfactant monolayer is far more complex than ions at neat water surface; moreover, the available data for the electrolyte adsorption are scarce and not systematic. Therefore, it should not be a surprise that the ion-monolayer interactions are not well understood. We will nevertheless highlight some features that appear to be general.

### Equilibrium Spread Monolayers

**Figure 9:**
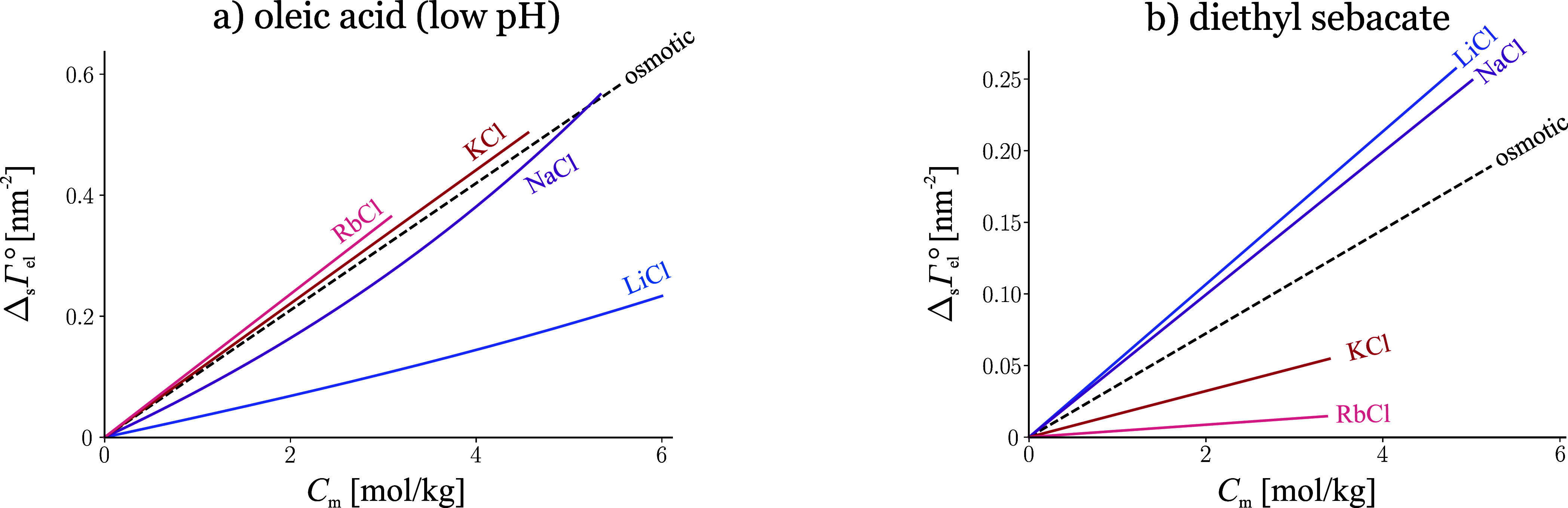
Monolayer-induced adsorption on equilibrium spread monolayers of liquid lenses of a) undissociated oleic acid and b) diethyl sebacate;^17^ based on *π*° data from refs (106) and (107) and eq 32. The line “osmotic” stands for eq 40 (no specific interactions). Adapted from ref (17). Copyright 2023 ACS.

In ref (17) we extracted the electrolyte adsorption from published data^106,107^ for the equilibrium spreading pressure *π*° of lenses of oleic acid and diethyl sebacate on various electrolyte substrates. Figure 9 compares the adsorption of several chlorides on dense monolayers of undissociated oleic acid and diethyl sebacate. Both amphiphiles produce positive monolayer-induced adsorption, more so the acid: e.g., at 4.5 mol/kg KCl, we have found Δ_s_Γ_el_ ∼ + 0.5 nm^–2^ on the acid vs Δ_s_Γ_el_ ∼ + 0.05 nm^–2^ on the ester (compared to Γ_el_ = −0.85 nm^–2^ at neat W|A, see Figure 5a). The absolute electrolyte adsorption remains negative in all cases. The osmotic effect appears to be the dominant reason for the extra adsorption of NaCl, KCl and RbCl on oleic acid–these salts follow approximately the osmotic isotherm (eq 38); LiCl, however, is repulsed by the acid monolayer. The data for oleic acid appear to be sensitive enough to capture a direct ion-specific effect: the larger the ion the more it adsorbs. The opposite situation is observed with diethyl sebacate: the smaller the ion the more it adsorbs. The “baseline” in the case of the ester monolayer must be viewed with caution as the size of the headgroup is too large for the model (40) to hold–we use it only to give an idea about the order of magnitude of the osmotic effect; *V*_s_^*ε*^ is calculated assuming headgroup having volume and polarizability like HCOOC_2_H_5_ in its liquid phase.

The data in Figure 9 are an example of lyotropic series reversal: the direct cation series is followed with the more polar —COOH head groups (Rb^+^ adsorbs most), while the reverse order is observed with the less polar —COOC_2_H_5_ (Li^+^ adsorbs most). The behaviors at both monolayers differ from the adsorption of the same salts at surfactant-free W|A and W|H where SchM is followed^9,12^ (see Figure 4a, Figure 5a, Figure 6a for examples; the only exception is LiCl above 3 mol/kg). These results agree with the maps from ref (111) (based on MD): at zero surface charge, for a —CH_3_ terminated surface, Na^+^ is predicted to adsorb more than K^+^; at —OH-terminated surface, K^+^ adsorbs more; in both cases, large anions adsorb more (I^–^ > Cl^–^ > F^–^^17^).

### Langmuir Trough: Γ_el_ vs Surfactant Area per Molecule

If the adsorptions of surface inactive ions at both surfactant-free surfaces (Γ_s_ = 0) and at surfaces with densely packed monolayer (Γ_s_ = Γ_s_^°^) exhibit behavior dominated by dehydration and image forces, then it may be expected that SchM would be reasonably accurate for electrolytes on half-filled monolayer (Γ_s_ ∼ 1/2Γ_s_^°^). This expectation is consistently denied by the experimental data. Instead, there usually appears a pronounced maximum of the attraction of the electrolyte to the monolayer surface at a certain intermediate density.

**Figure 10:**
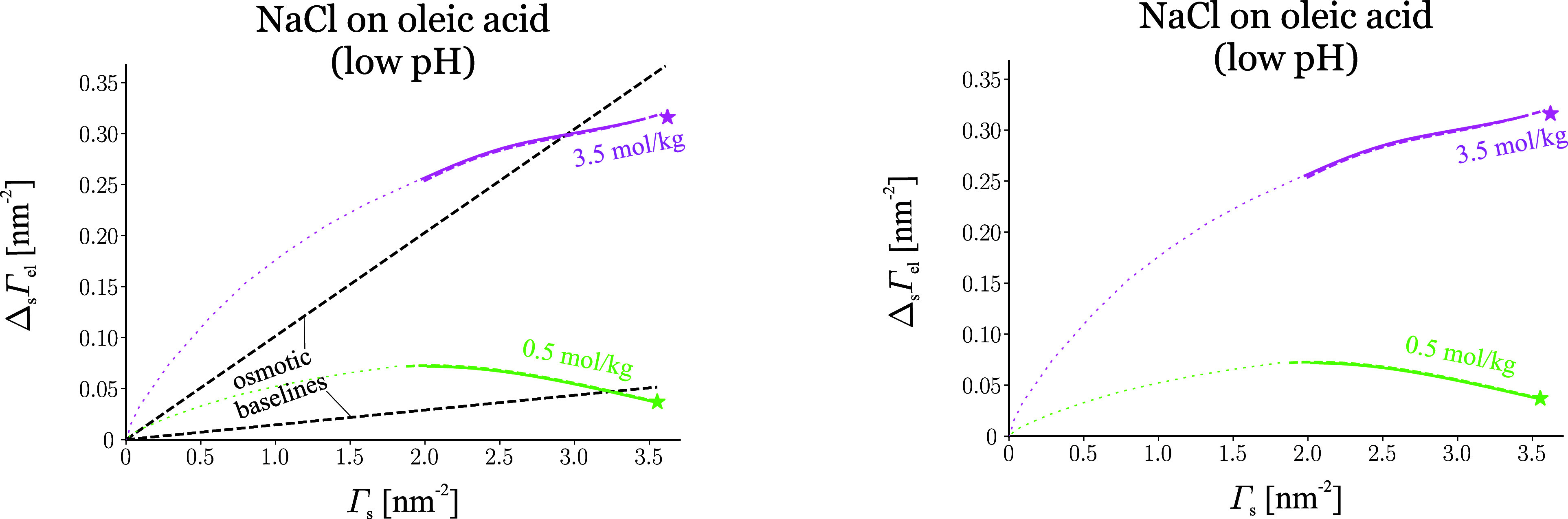
NaCl adsorption on uncharged monolayer of oleic acid of varying monolayer density Γ_s_,^17^ based on data from ref (107). The lines 0.5 and 3.5 mol/kg are calculated via numerical differentiation through eq 34, with Δ*μ*_s_ calculated through eq 33. The dotted line is an extrapolation: the curve must start from the origin, according to the definition of Δ_s_Γ_el_, eq 39. The osmotic baselines stand for eq 40 (no specific interactions). Stars: adsorptions calculated straight from equilibrium spreading pressure of oleic acid lenses via eq 32. Adapted from ref (17). Copyright 2023 ACS.

An example is given in Figure 10, with NaCl adsorbing on a liquid expanded monolayer made of oleic acid at low pH. The osmotic effect (eq 40) is again contributing significantly to the ion-monolayer interaction (the osmotic baseline in Figure 10). However, at higher compression, there is a tendency for the ions to be expelled by the monolayer, and to be attracted by it at coverages of ∼50%. Similar maximum is displayed by LiCl.^17^

**Figure 11:**
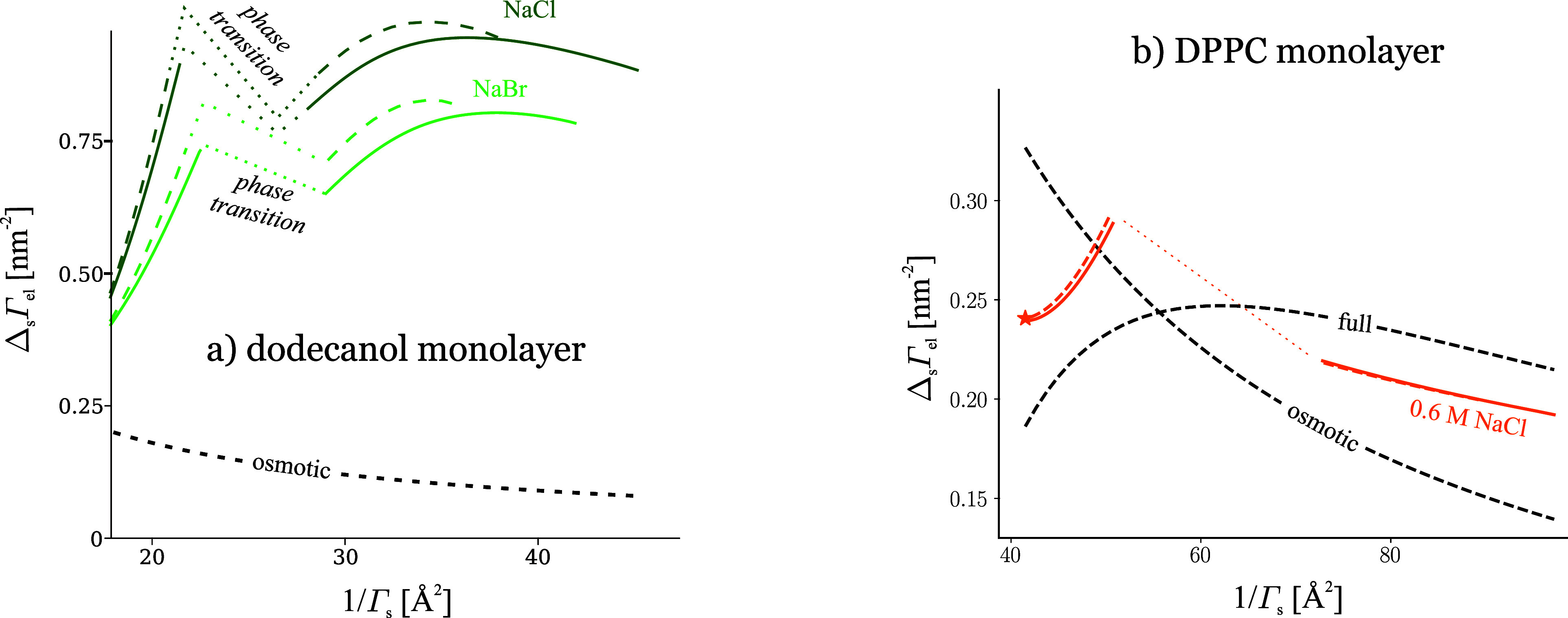
a) Monolayer-induced adsorption of 2 mol/kg NaCl and NaBr at dodecanol monolayer of varying area per molecule. Adapted from ref (102). Copyright 2016 ACS. b) Same for 0.6 M NaCl on DPPC (calculated from compression isotherms from ref (112)). Adapted from ref (109). Copyright 2023 ACS.

The picture becomes more intricate with dodecanol, which makes a phase transition from 2D liquid to 2D solid state. The adsorption of NaCl and NaBr is discontinuous at the phase transition point,^102^ like the surfactant adsorption Γ_s_: at the two-phase coexistence point, the 2D solid surfactant phase attracts the electrolyte more strongly. Also, the 2D solid exhibits a steeper slope dΔ_s_Γ_el_/dΓ_s_, eventually becoming more repulsive than the 2D liquid, Figure 11a. While this complicates the Δ_s_Γ_el_ vs Γ_s_ isotherm, it is still true that the salt adsorption exhibits a pronounced maximum at a certain intermediate coverage (∼50%) of the dodecanol monolayer. The maximum of Δ_s_Γ_el_ is ion-specific, with the smaller ions having higher affinity to the monolayer (NaF > NaCl > NaBr^102^).

The same experimental approach was used to determine the adsorption of NaCl on the zwitterionic phospholipid dipalmitoylphosphatidylcholine (DPPC),^109^Figure 11b. In this case, to calculate the osmotic baseline (40), we assume that the zwitterionic headgroup is very polar and we set its dielectric volume equal to that of phosphatidylcholine, *V*_s_^*ε*^ = *V*_head_ = 226 mL/mol. The shape of the Δ_s_Γ_el_ vs Γ_s_ isotherm is similar to that of dodecanol, with a phase transition, a maximum of the attraction, and with expulsion of ions from the monolayer at the highest densities.

The behavior demonstrated in Figure 10 and Figure 11 cannot be explained based only on the forces discussed in sec. Ions at surfactant-free W|A and W|H. From the fact that NaCl adsorbs similarly on neat W|A and W|H, Figure 6, it follows that the observed Δ_s_Γ_el_ vs Γ_s_ isotherm must be the result of a direct ion-surfactant headgroup interaction. The solvation of the ions at the surface incorporates the polar surfactant functional groups in the ion solvation shell, substituting water, possibly forming complexes with the ion. This process, electrolyte in the bulk + surfactant ⇄ electrolyte at the surface, corresponds to the following condition for equilibrium:
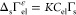
41

The salt-surfactant association constant *K* can include effects from direct complexation, ion-surface dipole interaction,^102^ and the diminished ion-surface dispersion interaction (a liquid monolayer neutralizes the dispersion similarly to a hydrocarbon phase, cf. Figure 6). Equation 41 coincides with the low-concentration limit of the isotherm proposed by Kralchevsky et al.^113^ for adsorption of ions on charged monolayers (they refer to it as “Stern isotherm”, but Stern never had the proportionality Δ_s_Γ_el_^*ε*^ ∝ Γ_s_, which is an important distinction, cf. Figure 10). However, attraction alone cannot explain the maximum of Δ_s_Γ_el_ – this requires additionally an expulsion of ions from the monolayer. One has to take into account the following effects:^109^

(i) The volume excluded by the head groups is not available for the electrolyte to occupy. For a monolayer of density Γ_s_, the area not occupied by surfactant is 1–0.91 × Γ_s_/Γ_s_^°^, where 0.91 is a packing correction (densely packed hexagonal lattice of discs has 9% of the area not covered by the discs). The head groups inhabit a layer of thickness *V*_head_Γ_s_^°^. At a surfactant-free surface, a layer of this thickness would contain *V*_head_Γ_s_^°^*C*_el_ moles of the electrolyte per unit area plus the negative surface excess Γ_el0_^*ε*^. Therefore, the surfactant monolayer excludes 0.91 × Γ_s_/Γ_s_^°^ × (*V*_head_Γ_s_^°^*C*_el_ + Γ_el0_^*ε*^) of the electrolyte, contributing negatively to Δ_s_Γ_el_^*ε*^.
(ii) For a geometry of the ion-surfactant associate where the ions are within the same layer of thickness *V*_head_Γ_s_^°^, the volume excluded by the head groups diminishes also the number (41) of ions associated with the surfactant from the unperturbed *KC*_el_Γ_s_ to *KC*_el_Γ_s_(1–0.91 × Γ_s_/Γ_s_^°^).

Combining these two effects with the osmotic effect, eq 39, we obtain what can be called the complexation-expulsion-osmotic (CEO) isotherm:
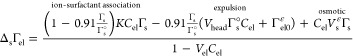
42

This isotherm has a single unknown: the value of the association constant *K*. Figure 11b illustrates the model with *K* = 0.42 M^–1^ (corresponding to one specifically adsorbed ion per about four phosphatidyl choline head groups at 0.6 M). The Γ_el_(*C*_m_,Γ_s_) data for NaCl on dodecanol is in reasonable agreement with eq 42 with *K* = 0.18 ± 0.04 M^–1^ in the whole studied range of areas and concentrations (1/Γ_s_ = 18–50 Å^2^, *C*_m_ = 0–2 mol/kg). For NaBr on dodecanol, *K* falls down to 0.08 ± 0.02 M^–1^. The higher affinity of NaCl compared to NaBr to the dodecanol monolayer (Figure 11a) can be explained with a relatively strong association of Na^+^ and Cl^–^ with the alcohol group in a dense monolayer while a bulky Br^–^ does not fit in the monolayer. Another possible reason is that the smaller ions show larger differences in solvation energy by water vs by surfactant headgroup.

For small head groups, the expulsion term is close to zero, and the association term in eq 42 has a maximum at coverage Γ_s_/Γ_s_^°^ = 0.55, explaining the observed location of the maximum of ion-monolayer attraction for acids and alcohols. The agreement with the data can be improved if two phase-specific values of *K* are allowed for, or by putting more detail into the expulsion model. However, the data in the 2D solid region are quite uncertain due to the slow kinetics of equilibration of the solid monolayer upon compression;^102^ therefore, the CEO model is currently sufficiently accurate in its form (eq 42).

An interplay between complexation and volume exclusion effects was first proposed by Leontidis and Aroti.^114^ The two effects have been observed in MD studies of electrolyte-phospholipid systems. For example, Gurtovenko and Vattulainen^115^ and Cordomí et al.^116^ reported Na^+^ binding to the phospholipid headgroup while the larger K^+^ is excluded from the surface. There is little agreement about the magnitude of the ion-lipid association; for example, Catte et al.^117^ reported that most force fields overestimate the cation binding and were sceptical about the reality of the specific binding of the ions to the phospholipid. MD simulations that take into account the ion polarizability, however, produce a weak binding of Na^+^ to the lipid.^118,119^ In any case, we are not aware of systematic studies of the adsorption of electrolyte on monolayers of varying density, and Figure 10 and Figure 11 show that a 10–20% change in the monolayer compression is sufficient to change the situation from ion sticking to the surfactant to ions expulsed from the surface.

### Δ*V* Potential of Monolayers on Saline Solutions

The Δ*V* potential is the change of the surface potential upon spreading a monolayer at a surface:
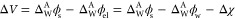
43

Here, Δ_W_^A^*ϕ*_s_ ≡ Δ_W_^A^*ϕ*(Γ_s_,*C*_el_) is the surface potential of a monolayer spread on an electrolyte solution, and Δ_W_^A^*ϕ*_el_ ≡ Δ_W_^A^*ϕ*(Γ_s_ = 0,*C*_el_) is surface potential of the surfactant-free surface of the solution; we used eq 25 for Δ_W_^A^*ϕ*_el_ in order to relate the monolayer’s Δ*V* potential to the surface potential of pure water, Δ_W_^A^*ϕ*_w_ ≡ Δ_W_^A^*ϕ*(Γ_s_ = 0,*C*_el_ = 0). Equation 23 that we used for Δ_W_^A^*ϕ*_el_ holds also for Δ_W_^A^*ϕ*_s_, but *P*_s_ and *ϕ*_s_^DL^ in the presence of surfactant are different;^102^ from eq 43:
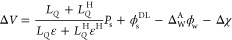
44

Note that if surfactant monolayer is present at the W|A surface, one cannot set *L*_*Q*_^H^ = 0: the monolayer is thicker (∼10 Å) than the characteristic decay length (*L*_*Q*_^H^ ∼ 1 Å) of the field created by the adsorbed dipoles. The quadrupolar length *L*_*Q*_^H^ is, in this case, characterizing the quadrupolar response of the hydrocarbon tail region, and there is a diffuse dipolar layer not only in the aqueous substrate but also in the hydrophobic liquid-like film of alkyl chains above the dielectric surface. The aqueous phase is more polar and *ε* ≫ *ε*^H^ in eq 44, but the quadrupolar lengths *L*_*Q*_ and *L*_*Q*_^H^ are expected to be of a similar order of magnitude,^23,96,97^ i.e.:
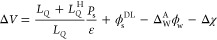
45

The same equation can be written for a monolayer on pure water (*C*_el_ = 0):
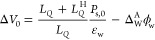
46

Thus, in the presence of surfactant, the structure of the dipolar double layer is again essential: the medium response to the field of the surface condenser *P*_s_ is nonlocal. The empirical local surface permittivity *ε*^S^ appearing in the Helmholtz model^120,121^ should actually be substituted with *L*_*Q*_/(*L*_*Q*_ + *L*_*Q*_^H^) × *ε*.

The comparison between eqs 45 and 46 shows that, similarly to the Δ*χ* potential, the change in Δ*V* of an uncharged monolayer with the electrolyte concentration is the result of three effects: **(i)** the formation of an EDL by the electrolyte (through *ϕ*_s_^DL^ and Δ*χ*); **(ii)** the effect of the ions on the adsorbed dipole moment, changing *P*_s,0_ to *P*_s_, via mechanisms similar to eqs 26-28 but now involving also the dipole carried by the surfactant head groups; **(iii)** the indirect ion-specific effect through the bulk dielectric permittivity (*ε* of the salt solution is smaller than *ε*_w_ of pure water).

One of these three effects is straightforward to confirm: the value of Δ*V* at a fixed monolayer density has been shown^17^ to be indeed strongly correlated to the ion-specific value of the dielectric permittivity *ε*. The sticky ions are outliers from this correlation; e.g., the addition of KI makes the Δ*V* potential of hexadecanol and ethyl palmitate monolayers much more positive, in the opposite direction of the effect from *ε* and even though the value of *ϕ*^DL^ is expected to be negative. As noted by Frumkin and Pankratov,^104,105^ this indicates that I^–^ penetrates the monolayer and alters dramatically the dipolar contribution to Δ*V*. This is important to highlight as it has been a widespread practice in recent years to interpret changes in Δ*V* with the electrolyte concentration as the result of the EDL potential alone. This is erroneous. Actually, for surface inactive electrolytes, *ϕ*_s_^DL^ is often canceled in eq 45 by the *ϕ*^DL^ term for surfactant-free surface that appears in eq 25 for Δ*χ*.

It is not currently possible to disentangle the effects from the change with *C*_el_ of *P* and the one from the EDL potential in eq 45: both appear to be significant. Two possible limiting cases can be investigated: **(i)***P*_s_ = *P*_s,0_ (a monolayer of a fixed density has a fixed specifically adsorbed dipole), which allows to calculate *ϕ*_s_^DL^ from Δ*V*; and **(ii)***ϕ*_s_^DL^ = *ϕ*^DL^ (the monolayer has negligible effect on the EDL), which allows to calculate *P*_s_ from Δ*V*. Such analysis is presented in refs (17) and (102) and is useful for bracketing the electric effects occurring in the monolayer.

## Conclusions

### Surfactant-Free Surfaces

We equipped Schmutzer’s theory for the adsorption of electrolytes^20^ with explicit models for the dehydration force (eqs 12 and 13, using data for the structure of the hydration shell), for the surface layer (Figure 2), and for the EDL (Figure 3). The final formula for the electrolyte adsorption, eqs 14-16, is a useful tool to subtract from tensiometric and potentiometric data the effect from the better understood image and dehydration ion-surface interaction, together with the indirect effect from the ion-specific electrolyte activity coefficient, and thus to elucidate the nature of the direct specific ion-surface interactions. This approach allowed us to conclude that

(i) the typical sticky ions (I^–^, SCN^–^) are attracted to W|A by a hydrophobic attraction counteracted by repulsive VdW force (combining a larger repulsive dispersion and smaller attractive static polarization contributions). These ions stick better to W|H, where the dispersion is switched off. The stickiness appears above a certain threshold concentration *C*_thr_ (Table 1); even iodides behave as nonsticky at ∼0.5 mol/kg, see Figure 7. The value of *C*_thr_ correlates strongly with the ion size.
(ii) For the borderline ions (Br^–^, NO_3_^–^), the contributions of the hydrophobic and VdW forces cancel in the adsorption; however, due to the dispersion repulsion being longer ranged, these interactions still show up in the surface potential data (see the supplement). Despite the popular opinion, the available Δ_el_*σ* data suggest that alkali bromides and nitrates have a more negative adsorption than the respective chlorides at W|A, at least up to *C*_thr_ = 2.5–6 mol/kg; the combination of specific forces cause polarization without net adsorption.
(iii) For most surface inactive ions, SchM agrees with the available Δ_el_σ and Δχ data. These ions still exhibit ion-specific surface tension and surface potential, via the indirect effects of the bulk activity coefficient and dielectric permittivity, respectively (Figure 5 and Figure 8).
(iv) Several other significant effects have been identified for certain ions: there are clear effects of the permanent dipole of ions like HCOO^–^, ClO_3_^–^, OH^–^, H_3_O^+^ on Δ_el_*σ* and Δ*χ*; highly charged 2:2, 3:1 and 3:2 electrolytes are exhibiting Wagner’s effect (extra repulsion due to negative excess conductivity in the surface layer, a second-order ion correlation effect); H_3_O^+^ and NH_4_^+^ fit better in the hydrogen bond network at the surface than in the bulk; a number of ion–ion and ion–water association effects etc.

### Adsorption of Ions at Uncharged Monolayers

The monolayer-induced adsorption Δ_s_Γ_el_ (the extra adsorption on top of Γ_el,0_ at the surfactant-free surface) is characterized by an adsorption isotherm, Δ_s_Γ_el_(*C*_m_,Γ_s_), which is a function of the electrolyte concentration and the monolayer density simultaneously.

(i) We were able to determine such isotherms from experimental data for insoluble uncharged monolayers, using an idea of Frumkin: to combine compression isotherms with data for the equilibrium spreading pressure of the surfactant phase (Figure 10-Figure 11). The isotherm is sensitive to the nature of the headgroup, the state of the surface (ions adsorb differently on 2D liquid and 2D solid monolayers) and is ion-specific even for ions that are surface inactive at W|A. The experimental dependence of Γ_el_ on Γ_s_ suggests that a maximum of the ion-monolayer attraction appears for monolayers of intermediate density.
(ii) The main effects producing the extra adsorption Δ_s_Γ_el_ on monolayers appear to be an osmotic effect from the dilution of the surface layer by the surfactant polar groups; specific ion-surfactant association (probably a combination of solvation, VdW and ion-surface dipole interactions), and a volume exclusion effect (steric ion-polar headgroup interaction).

Putting these effects together results in the CEO isotherm, eq 42. This equation is bridging the adsorption Δ_s_Γ_el_ caused by the monolayer to the adsorption at the surfactant-free surface (through Γ_el0_^*ε*^). Equation 42 has a form similar to the isotherm of Kralchevsky et al.^113^ developed for charged monolayers, so the CEO model is possibly applicable to ionic surfactants, by substituting the electrolyte concentration with Σ*C*_*i*_exp(−*e*_*i*_*ϕ*^DL^/*k*_B_*T*).

## Supporting Information Available

List of symbols and abbreviations and polarization of the ion concentration profiles by the ion-specific interactions (PDF)
