# Supplementary material for: Electrolytes at Uncharged Liquid Interfaces: Adsorption, Potentials, Surface Tension, and the Role of the Surfactant Monolayer
Source: Langmuir. 2024 Aug 12;40(33):17170–89. doi: 10.1021/acs.langmuir.4c01388 (PMC11340029; doi:10.1021/acs.langmuir.4c01388)
Supplement: Supplementary file 1 — la4c01388_si_001.pdf [file la4c01388_si_001.pdf]

Electrolytes at uncharged liquid interfaces: adsorption, potentials, surface tension and the role of the surfactant monolayer

(supplementary information)

Radomir Iliev Slavchov[1*], Boyan Peychev[1,2], Ivan Minkov[2,3]

[1] School of Engineering and Materials Science, Queen Mary University of London, London E1 4NS, United Kingdom; corresponding author: r.slavchov@qmul.ac.uk
[2] Rostislaw Kaischew Institute of Physical Chemistry, Bulgarian Academy of Sciences, 1113 Sofia, Bulgaria
[3] Department of Chemistry, Biochemistry, Physiology, and Pathophysiology, Faculty of Medicine, Sofia University, 1407 Sofia, Bulgaria

List of symbols and abbreviations

Symbols:

C_{el} concentration of electrolyte
C_{m} molality of the electrolyte
C_{thr} threshold concentration above which the electrolyte behaves as sticky
E_1 exponential integral function
e elementary charge
K association constant (ion in the bulk + surfactant ⇄ ion-surfactant associate)
k_{B} Boltzmann constant
$k_{\mathrm{is}} = (\nu_+ Z_+^2 + \nu_- Z_-^2)/2$ ratio between ionic strength and electrolyte concentration C_{el}
L_{D} Debye length, a ratio between dipolar and ionic strength, $L_{\mathrm{D}}^2 = \varepsilon k_{\mathrm{B}} T / 2 k_{\mathrm{is}} e^2 C_{\mathrm{el}}$
L_Q quadrupolar length, a ratio between quadrupolar and dipolar strength
N_{w} water molecules in the surface layer fully orientated by an ion in the subsurface layer
n_{w} refractive index of water
P specifically adsorbed normal surface dipole moment
p mean normal dipole moment
R_i minimum distance of approach of an ion to the surface $z = 0$; $R_i = R_{\mathrm{h},i} - z_{\mathrm{h}}$
$R_{\mathrm{bare},i}$ radius of the bare i^{th} ion
$R_{\mathrm{h},i}$ radius of the hydration shell of the ith ion
R_{w} radius of a water molecule
T temperature
$u_{\mathrm{h},i}$ dehydration potential acting on the ith ion
$u_{\mathrm{im},i}$ image potential acting on the ith ion
V_{el} partial molecular volume of the electrolyte
V_{w} partial molecular volume of water
$\Delta V = \Delta_{\mathrm{W}}^{\mathrm{A}}\phi_{\mathrm{s}} - \Delta_{\mathrm{W}}^{\mathrm{A}}\phi_{\mathrm{el}}$ Volta potential change upon spreading a monolayer

ΔV_0 Volta potential change upon spreading a monolayer on pure water
x density of the surface layer of molecules contributing to P divided by density of bulk
Z_i nondimensionalized absolute charge (valence) of the ith ion, $Z_i = |e_i|/e$
$z = 0$ location of the ε-discontinuity
z_w shift of water's equimolecular surface with respect to the ε-discontinuity surface
z_h thickness of the hydrophobic gap

α_i molecular or ionic polarizability
Γ_el electrolyte adsorption (surface excess of C_el)
Γ_p the number of water dipoles contributing to P per unit area
Γ_s adsorption of the surfactant ($1/\Gamma_\mathrm{s}$ is area per molecule)
Γ_w adsorption of water
γ_el molality-based mean activity coefficient
ε absolute dielectric permittivity of the solution
ε_0 absolute dielectric permittivity of vacuum
ε_w absolute dielectric permittivity of pure water
μ_el chemical potential of the electrolyte
μ_s chemical potential of the surfactant
ν isotonic coefficient, $\nu = \nu_+ + \nu_-$
ν_i stoichiometric number of the ith ion
π^S surface pressure of a monolayer, $\pi^\mathrm{S} = \sigma_0 - \sigma$
σ surface tension
σ_0 surface tension of the electrolyte solution in the absence of surfactant
σ_w surface tension of pure water
$\Delta_\mathrm{el}\sigma = \sigma_0 - \sigma_\mathrm{w}$ surface tension increment upon addition of electrolyte
ϕ^DL potential of the EDL
$\Delta_\mathrm{W}^\mathrm{A}\phi$ surface potential
$\Delta_\mathrm{W}^\mathrm{A}\phi_\mathrm{el}$ surface potential of the electrolyte solution in the absence of surfactant
$\Delta_\mathrm{W}^\mathrm{A}\phi_\mathrm{s}$ surface potential with both electrolyte and surfactant
$\Delta_\mathrm{W}^\mathrm{A}\phi_\mathrm{w}$ surface potential of pure water
$\Delta\chi = \Delta_\mathrm{W}^\mathrm{A}\phi_\mathrm{el} - \Delta_\mathrm{W}^\mathrm{A}\phi_\mathrm{w}$ surface potential change upon addition of electrolyte (in direction of air)

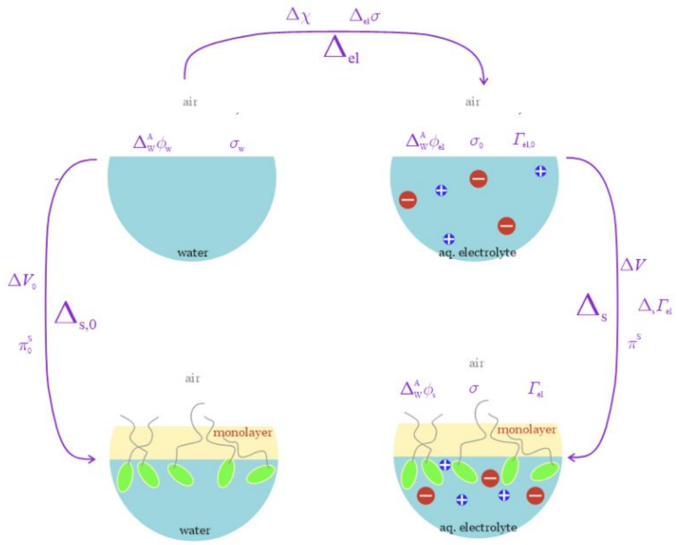

Figure S1. Symbols used to indicate surface potential, surface tension, electrolyte adsorption at various kinds of surfaces, and symbols used for the change of these quantities upon adding electrolyte or surfactant monolayer.

Abbreviations:

DDL dipolar double layer
EDL electric double layer
SchM modified Schmutzer's model (the minimal model of choice)
X_0 referring to surface without surfactant but with electrolyte
X_h referring to dehydration
X_im referring to image force
X_w referring to water
X^ε referring to the surface of dielectric discontinuity
X° referring to the pure surfactant phase / the equilibrium spread monolayer around a crystal or a droplet of the surfactant phase
W|A water|air surface
W|H water|hydrocarbon interface
W|M water|air surface with a surfactant monolayer

Polarization of the ion concentration profiles by the ion-specific interactions

Figure S2 illustrates schematically the profile of Br^- at W|A and W|H interfaces (the effect from the EDL and the image forces is ignored for simplicity, making the schematic valid for very high concentration). At W|H, the dispersion repulsion is absent due to the similar Hamaker constants of the two phases. The hydrophobic and Debye forces are both attractive, producing some excess of bromide at the surface and negative deviations from SchM. The surface is negatively charged, and an EDL will be formed (Na^+-dominated diffuse layer electroneutralizing the surface).

In contrast, at W|A, water attracts the ions with a long-ranged dispersion force; the shorter ranged hydrophobic and (the screened) Debye forces are of similar magnitude as for W|H. The excess of Br^- is now zero, and no surface charge will accumulate. However, the surface is significantly polarized due to the specific forces alone, with negative excess charge in the layer dominated by the hydrophobic force, and positive charge in the dispersion force-dominated layer below. The cation will modulate the polarization but no EDL in the classical sense will form.

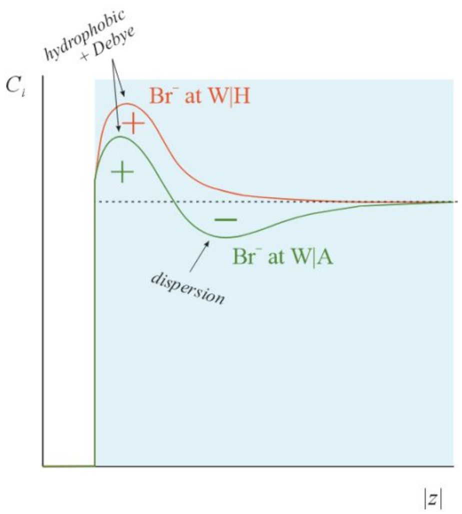

Figure S2. Schematic ion concentration profile for Br^- at W|H (where it adsorbs) and W|A (where concentration polarization appears without significant adsorption).
